# Discovery of ONO-TR-772 (VU6018042): A Highly Selective and CNS Penetrant TREK Inhibitor *in Vivo* Tool Compound

**DOI:** 10.1021/acsmedchemlett.5c00215

**Published:** 2025-04-28

**Authors:** Motoyuki Tanaka, Takahiro Mori, Gakuji Hashimoto, Katsukuni Mitsui, Akihiro Kishi, Elizabeth S. Childress, Sean R. Bollinger, Trevor C. Chopko, Thomas M. Bridges, Douglas G. Stafford, Zhonping Huang, Mark A. Wolf, Darren W. Engers, Jerod S. Denton, Haruto Kurata, Craig W. Lindsley

**Affiliations:** † Drug Discovery Chemistry, 13369 Ono Pharmaceutical Co., Ltd, 3-1-1 Sakurai, Shimamoto, Mishima, Osaka 618-8585, Japan; ‡ Research Center of Neurology, Ono Pharmaceutical Co., Ltd, 3-1-1 Sakurai, Shimamoto, Mishima, Osaka 618-8585, Japan; § Warren Center for Neuroscience Drug Discovery, Vanderbilt University, Nashville, Tennessee 37232, United States; ∥ Department of Pharmacology, 5718 Vanderbilt University School of Medicine, Nashville, Tennessee 37232, United States; ⊥ 17451 Curia Global, Inc., 24 Corporate Circle, Albany, New York 12203, United States; # Department of Anesthesiology, Vanderbilt University Medical Center, Nashville, Tennessee 37232, United States

**Keywords:** TWIK-related K+ channel (TREK), two-pore domain potassium channel (K_2_P), Novel object recognition (NOR), cognition, ion channel

## Abstract

Herein we describe our continuing work on the K_2_P family of potassium ion channels with the chemical optimization of a selective and CNS penetrant series of TREK inhibitors, culminating in the discovery of ONO-TR-772 (VU6018042). From an HTS hit harboring a benzyl ether linker, SAR proved intractable until an acetylene linker was identified as an isosteric replacement. Robust SAR was then observed, and a key fluorination to enhance PK and CNS penetration provided ONO-TR-772 (VU6018042), a potent (TREK-1 IC_50_ = 15 nM), selective (>10 μM versus other K_2_P channels except TREK-2), and CNS penetrant (rat *K*_p_ = 0.98) TREK inhibitor. ONO-TR-772 (VU6018042) demonstrated robust efficacy in an MK-801 challenge NOR paradigm, with an MED of 10 mg/kg.

## Graphical abstract

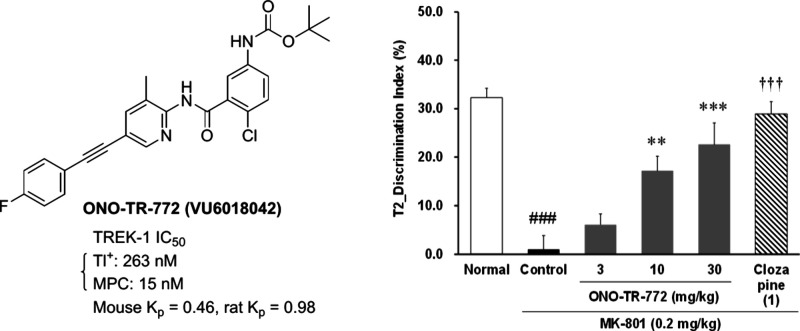

Potassium (K^+^) channels represent a major thrust of drug development activities targeting both shaker type voltage-gated (K_v_) and inward rectifier (K_ir_) channels.^1–6^ Far less explored is the third family, the two pore domain (K_2_P) family of potassium channels, consisting of 15 K_2_P subtypes, within six distinct subfamilies: tandem of P domains in a weakly inward rectifying potassium channel (TWIK), TWIK related K^+^ channels (TREK), TASK (TWIK related acid-sensitive K^+^ channels), TWIK related ALkaline pH-activated K^+^ channels (TALK), tandem pore domain halothane inhibited K^+^ channels (THIK), and TWIK related spinal cord K^+^ channel (TRESK).^1–6^ Our first priority was to develop potent and selective *in vivo* tool compounds to modulate TREK-1. TREK-1 is highly expressed in the central and peripheral nervous systems, and modulation of these channels are proposed to play key roles in multiple CNS and peripheral disorders; unfortunately, a deeper understanding of the therapeutic potential of TREK-1 has been hampered by a lack of small molecule tools.^1–12^

Recently (Figure 1), we disclosed ONO-2920632 (**1**, VU6011887),^13^ a highly selective and CNS penetrant TREK-2 preferring activator (structurally distinct from BL-1249 (**2**)^14,15^ ), with potential for nonopiate pain management. Attention now turned to the development of TREK inhibitors for the potential treatment of cognitive deficits, as numerous studies implicate TREK-1.^7–12^ For example, inhibition of TREK-1 protects mice from cognitive impairment induced by anesthesia,^7^ and TREK-1 gene expression is increased in the hippocampus of schizophrenic patients relative to normal controls.^16^ Likewise, inhibition of TREK-2 is required for the neurotensin-mediated facilitation of spatial learning, and TREK-2 expression is increased in the cortex and hippocampus of several CNS pathologies.^17,18^ Taken together, these data argue for the need to pharmacologically validate the role of TREK-1 and TREK-2 inhibition in preclinical rodent cognition models. Yet, the tools to do so are limited to peptides such as spadin,^19^ weak (IC_50_s 2 to >10 μM) off-target small molecule inhibitors such as fluoxetine,^20^ atypical antipsychotics,^21^ antihypertensives,^22^ and antiarrhythmic drugs.^23^ A state-dependent TREK-1 inhibitor, TKIM (**3**), has been reported with an IC_50_ of 2.96 μM^24^ as well as more recently the CNS-penetrant isobenzofuran-1­(3*H*)-one derivative, Cpd8 (**4**) (TREK-1 IC_50_ of 810 nM), which displayed selectivity versus TREK-2 and neuroprotective effects *in vitro* and *in vivo*.^25^ In this letter, we describe our efforts toward the development of a potent, selective, and CNS penetrant TREK inhibitor derived from a weak HTS hit.

**Figure 1:**
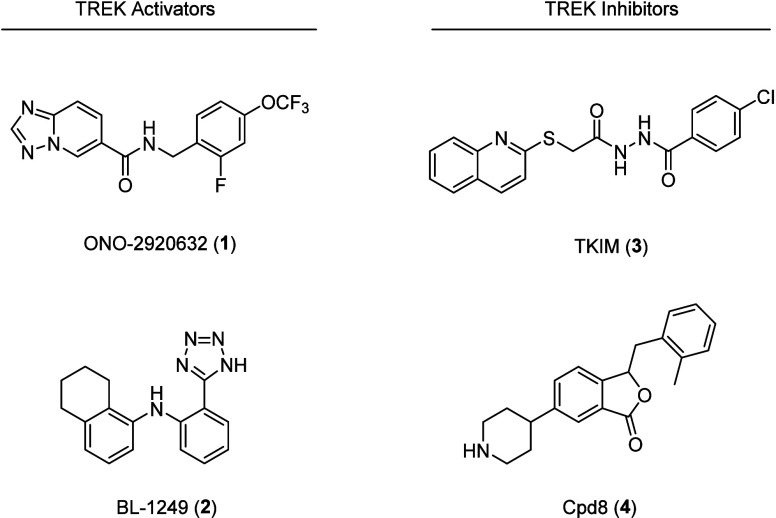
Structures of reported TREK-1 and TREK-2 activators **1** and **2**, and the more recently developed TREK-1 inhibitors **3** and **4**.

A high throughput screen employing a functional TREK-1 thallium (Tl^+^) flux assay identified (Figure 2) ONO-1530283 (**5**) as a weak TREK-1 inhibitor (IC_50_ = 7.7 μM). A hit expansion exercise increased TREK-1 potency ∼17-fold (IC_50_ = 0.46 μM) to afford ONO-0606822 (**6**), the lead for the program despite superhepatic rodent clearance and high clogP (5.2). Moreover, we were concerned about the latent quinone moiety in **6**; thus, the initial aim of the optimization was to remove the latent quinone by the addition of heteroatoms to the phenyl core while also reducing lipophilicity.

**Figure 2:**
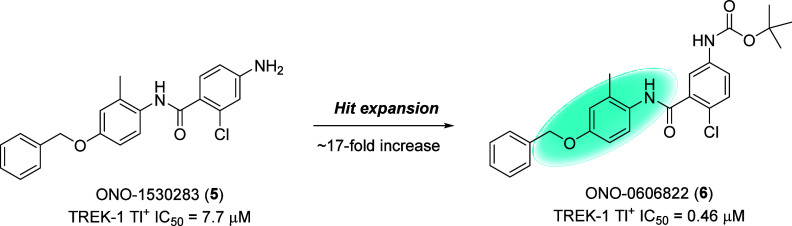
Structures of the HTS hit **5**, and a rapid hit expansion exercise improved potency ∼17-fold to afford submicromolar TREK-1 lead **6**.

Scheme 1 highlights the routes to access key heteroaryl congeners **10** of TREK inhibitor lead **6**. For both pyridine isomers and the pyrazine analogue, commercial starting materials **7**–**9** underwent either alkylation reactions or a Mitsunobu reaction to install the benzyl ether moiety, followed by nitro reduction, in the case of **8**, and a polyphosphonic anhydride (T_3_P) coupling with 5-(*tert*-butoxycarbonylamino)-2-chlorobenzoic acid to provide amide analogues **10**. TREK-1 inhibitory data is shown in Table 1; while the SAR was without texture, clogPs did decrease by almost a log (∼4.4), but both *in vitro* and *in vivo* clearance in rat remained suprahepatic (CL_p_ > 100 mL/min/kg). The *N*-Boc moiety proved to be, surprisingly, not responsible for the poor PK. However, these data prompted the team to modify the core for subsequent optimization to the pyridine congener **10a**.

**Scheme 1:**
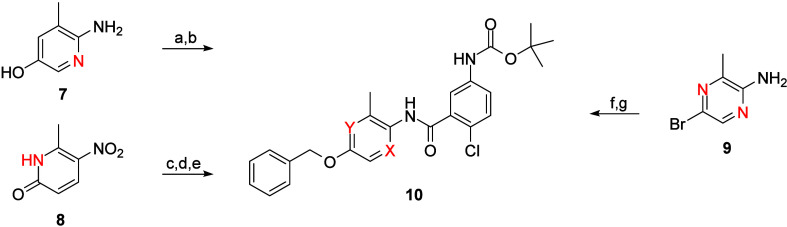
Synthesis of Heteroaryl Analogues **10** ^a^

a Reagents and conditions: (a) BnBr, Cs_2_CO_3_, DMF, rt, 12%; (b) 5-(*tert*-butoxycarbonylamino)-2-chlorobenzoic acid, DIEA, DMF, HBTU, rt to 60 °C, 18%; (c) BnOH, DIAD, PPh_3_, THF, rt, 24%; (d) Fe, NH_4_Cl, THF, H_2_O, 75 °C, 87%; (e) 5-(*tert*-butoxycarbonylamino)-2-chlorobenzoic acid, DIEA, DCM, T_3_P, rt, 42%; (f) BnOH, NaH, 0–120 °C, 34%; (g) 5-(*tert*-butoxycarbonylamino)-2-chlorobenzoic acid, DIEA, DCM, T_3_P, rt, 26%.

**Table 1:**
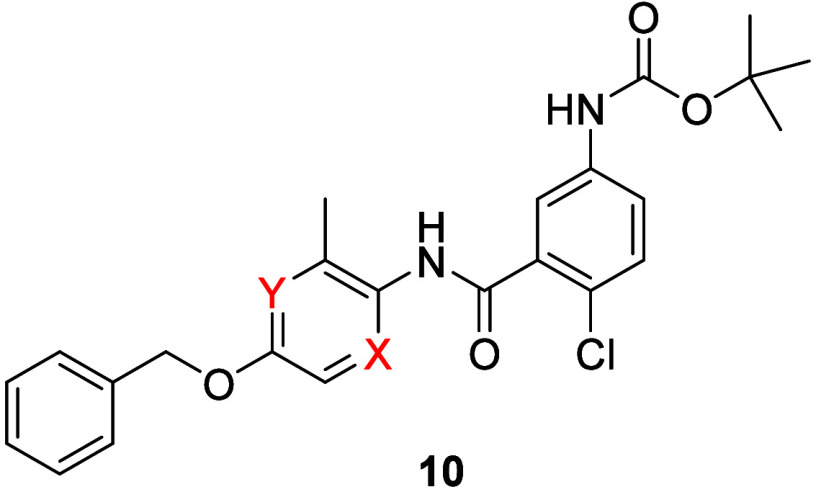

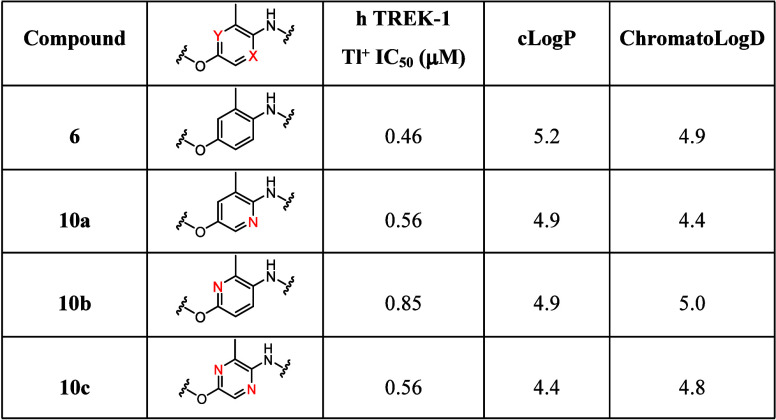
TREK-1 Inhibitory Activity and Lipophilicity of Analogues **10**

From previous work in our laboratories,^26^ we found that an acetylene was a productive bioisosteric replacement for a benzyl ether moiety, and we applied this tactic to both **10a** and **10c** (Figure 3) modifying Scheme 1 to replace the alkylation step with a Sonogashira coupling (see Supporting Information for full details) to provide **11** and **12**. In the case of **10a**, the acetylenic variant **11** displayed an ∼ 4-fold increase in TREK-1 potency (IC_50_ = 0.14 μM) and lowered human microsomal intrinsic clearance 10-fold (h CL_INT_ = 13 mL/min/kg). Interestingly, TREK-1 potency diminished in the pyrazine matched pairs **10c** (IC_50_ = 0.56 μM) and **12** (IC_50_ = 1.4 μM), but *in vivo* rat clearance improved dramatically with installation of the acetylene (**10c**: suprahepatic CL_p_= 135 mL/min/kg; **12**: CL_p_ = 25 mL/min/kg). Therefore, **11** became the new lead, and we elected to explore 2-position substituents on the pyridine core in analogues **13** (Table 2) as well as substituents on the distal phenyl ring pendant to the acetylene, as in derivatives **14** (Table 3).

**Figure 3:**
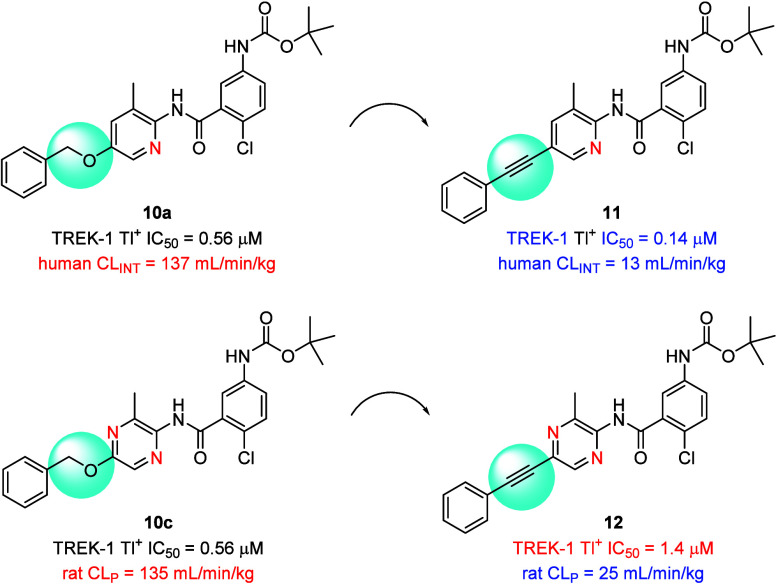
Impact of replacement of the benzyl ether with an acetylene moiety on both TREK-1 potency and *in vitro* and *in vivo* clearance.

**Table 2:**
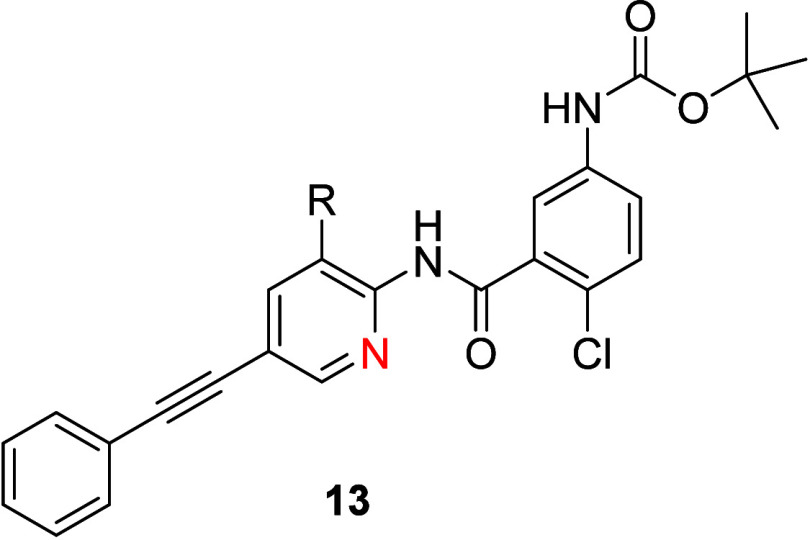
TREK-1 Inhibitory Activity and *in Vitro* DMPK Profiles of Analogues **13**

compd	R	h TREK-1 Tl^+^ IC_50_ (μM)	LMS CL_INT_ human/mouse (mL/min/kg)	PPB human/mouse (%)
**11**	Me	0.14	13/34	99.5/98.7
**13a**	F	0.11	10/6	99.8/98.9
**13b**	Cl	0.43	ND^a^	ND/>99.9
**13c**	OMe	0.91	ND	ND

a ND = not determined.

**Table 3:**
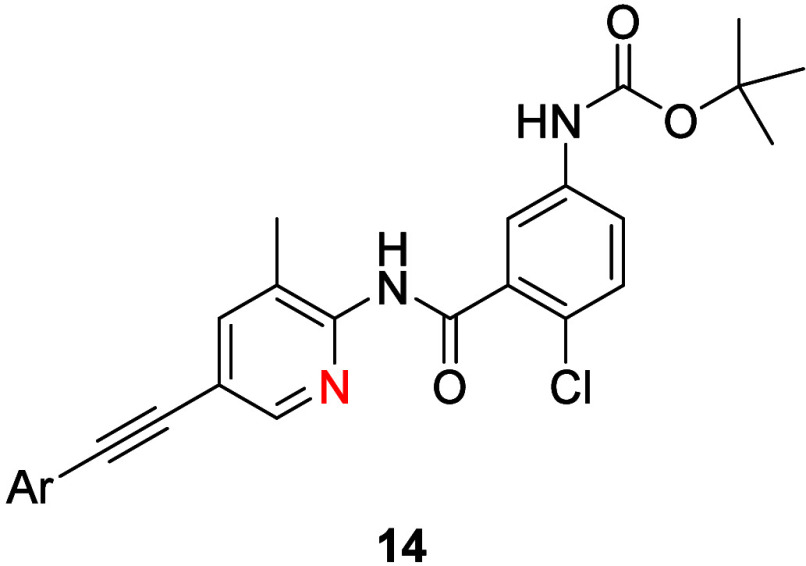

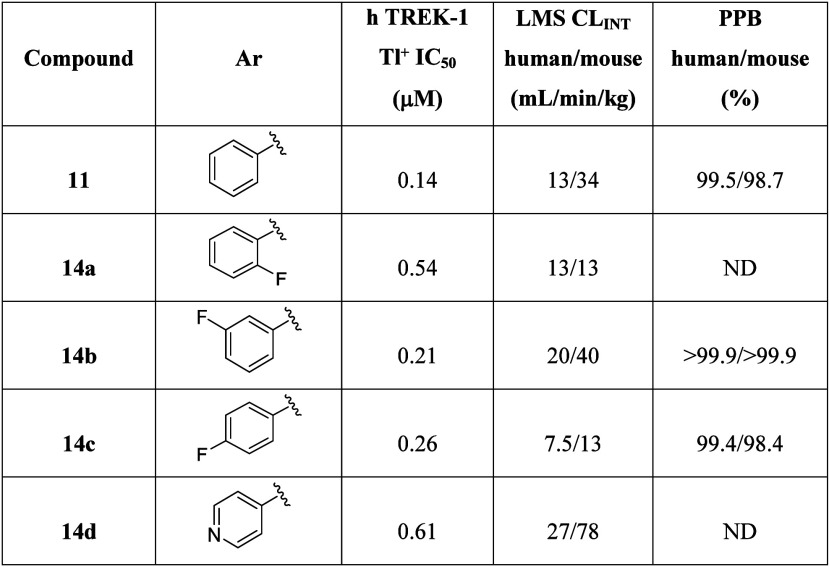
TREK-1 Inhibitory Activity and *in Vitro* DMPK Profiles of Analogues **13** ^a^

a ND = not determined.

In the 3-positon on the pyridine core (Table 2), a fluoro congener **13a** was equipotent to the parent methyl **6** and displayed similar or better microsomal stability and a little higher protein binding profiles. TREK-1 potency eroded as either a chlorine atom (**13b**, IC_50_ = 0.43 μM) was introduced or an electron-donating methoxy group (**13c**, IC_50_ = 0.91 μM). While the introduction of substituents to the distal phenyl ring of the acetylene moiety had no impact on TREK-1 potency (Table 3), a 4-F phenyl congener **14c** possessed improved microsomal stability (human CL_INT_ = 7.5 mL/min/kg, mouse CL_INT_ = 13 mL/min/kg). Thus, all efforts shifted toward a more detailed evaluation of **14c** (ONO-TR-772).

Up to this point in the program, SAR was guided by a thallium flux TREK-1 assay, it was now time to evaluate broader K_2_P and ion channel activity for **14c**. Gratifyingly, **14c** proved to be >67-fold selective over other K_2_P channels (TASK-3, TRESK, TWIK-2, TRAAK, TASK-1, TASK-2). Only TREK-2 was shown to be equipotent with TREK-1 in the profiling electrophysiological assays. In human manual patch clamp (MPC), **14c** was a potent TREK-1 inhibitor (IC_50_ = 15 nM), and therefore, more accurately described as a dual TREK-1/2 inhibitor. As mouse would be the species used in our cognition PD assay, we evaluated **14c** in mouse MPC, and it was a potent TREK-1 inhibitor (IC_50_ = 67 nM). Prior to performing cognition studies, we needed to assess broader ancillary pharmacology to ensure we would be evaluating selective TREK-1/2 inhibition. When gauged against a cardiovascular (CV) ion channel panel, **14c** was similarly highly selective (<50% at 10 μM versus: Ca_v_1.2, HNC4, K_ir_2.1, K_v_1.5, K_v_4.3, KCNQ1, Na_v_1.5 and hERG). The only activity >50% at 10 μM was Kir2.2 (52%). The clean ion channel pharmacology profile prompted the team to collect a broader ancillary pharmacology profile across GPCRs, ion channels and transporters in the Eurofins lead profiling screen. Here, **14c** showed >50% inhibition at 10 μM for only four of 68 targets: A_3_ (84%, *K*_i_ = 0.48 μM), Calcium L-type/benzothiazepine (56%, *K*_i_ = 2.6 μM), Calcium L-type/dihydropyridine (76%, *K*_i_ = 0.54 μM) and sodium channel, site 2 (84%, *K*_i_ = 0.65 μM). Weak activity at these targets would not obscure our proof-of-concept studies in rodent cognition models, so **14c** advanced into DMPK profiling.

In liver microsomes, CL_INT_ for **14c** was low across all species (h, r, m: 7.5, 29, and 13 mL/min/kg); however, the lipophilic nature of **14c** (clogP = 5.53) led to high plasma protein binding (h, r, m: 99.4%, 99.9% and 98.4%), brain homogenate binding (r, m: >99.9% and 99.4%) and low aqueous solubility (<5 μM). In a rat IV:PBL PK cassette study, **14c** demonstrated a good IVIVC with low clearance (CL_p_ = 21.2 mL/min/kg), high volume of distribution (6.7 L/kg) long half-life (*t*_1/2_ = 6.78 h) and a *K*_p_ of 0.98. In a 10 mg/kg PO mouse PBL study, **14c** displayed a *K*_p_ of 0.44, highlighting excellent CNS exposure in both mouse and rat; however, due to the high brain homogenate binding (driven by the lipophilic nature of **14c**), a *K*_p,uu_ could not be accurately ascertained. From the 10 mg/kg PO CD-1 male mouse study with **14c**, *C*_max_ total brain concentrations achieved 252 nM (*T*_max_ = 4 h, AUC 2269 h*nM), which we felt was too low to proceed with a novel object recognition (NOR) cognition assay. Switching to an IP route of administration (10 mg/kg) increased total brain exposure (*C*_max_ of 888 nM, 5.3 nM unbound based on mouse brain homogenate binding, *T*_max_ = 4 h, AUC 12,261 h*nM). Based on the lipophilicity of **14c**, we assumed the unbound brain level was a low estimate, so we elected to proceed with the NOR study at doses of 3, 10, and 30 mg/kg IP. Since the brain *T*_max_ was at 4 h, **14c** or the clozapine positive control was dosed IP to male CD-1 mice 3.5 h prior to the MK-801 (0.2 mg/kg IP) challenge, and then the discrimination index was assessed (Figure 4). As expected, MK-801 induced a significant cognitive deficit,^27^ which was dose dependently reversed by **14c**, with a minimum effective dose (MED) at 10 mg/kg and comparative efficacy at 30 mg/kg to the internal control clozapine (1 mg/kg IP). Thus, selective inhibition of TREK-1/2 was shown, for the first time, to be efficacious in the NOR paradigm and suggests therapeutic relevance for TREK-1 and or TREK-1/2 inhibition to treat cognitive disorders.

**Figure 4:**
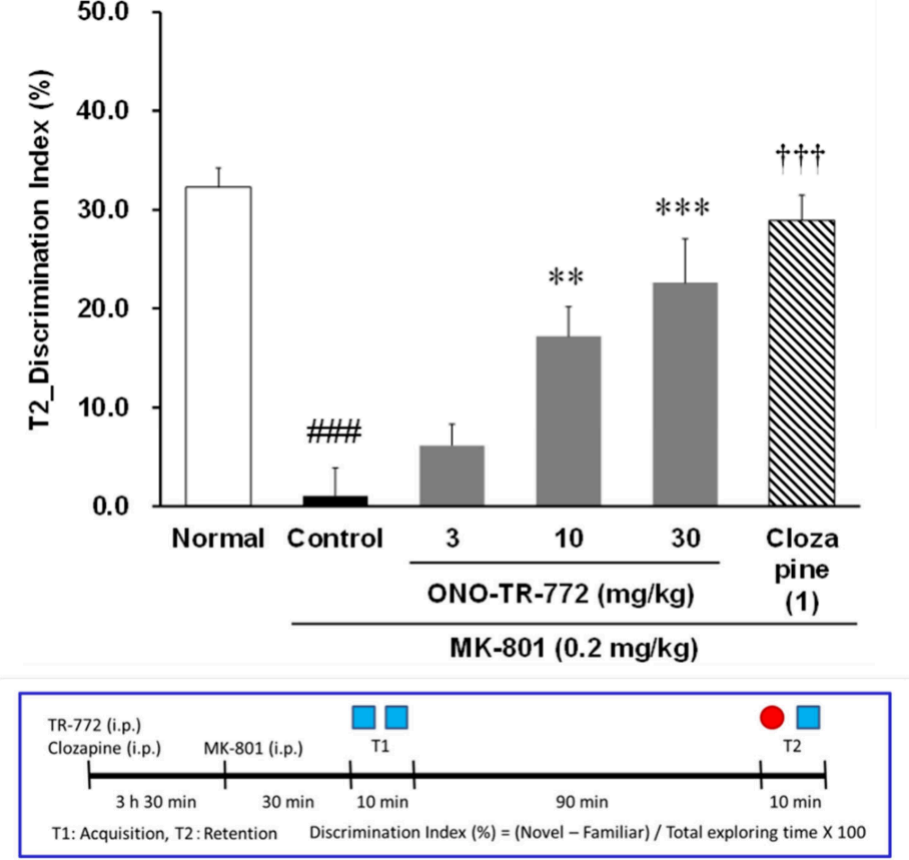
Effect of **14c** (ONO-TR-772/VU6018042) in the MK-801 challenged novel object recognition task. **14c** dose-dependently enhanced recognition memory in male CD-1 mice after challenged with MK-801. Pretreatment with 3, 10, and 30 mg/kg **14c** IP 3.5 h prior to MK-801 treatment and exposure to identical objects significantly enhanced recognition memory assessed 90 min later. Minimum effective dose (MED) is 10 mg/kg. *N* = 15/group of male CD-1 mice. # *p* < 0.05, ###, *p* < 0.001 vs normal (*t* test), **p < 0.01; ***p < 0.001 vs vehicle (Dunnett posthoc test). †††, *p* < 0.001 vehicle group (*t* test).

In parallel with the *in vivo* POC work, efforts were being made to find alternatives for the privileged *N*-Boc moiety, which contributed to the high lipophilicity and structural concern. Only one viable replacement emerged, a cyclopropyl amide (Figure 5), as shown in **15a** (TREK-1 IC_50_ = 0.18 μM) and **15b** (TREK-1 IC_50_ = 0.29 μM). In these matched pairs, the impact of the 4-F phenyl moiety was profound on both *in vitro* and *in vivo* clearance (10-fold reduction in *in vivo* rat CL_p_); however, both analogues still possessed high plasma protein binding (>99.7%) and no improvement on aqueous solubility. Further optimization on this series is required to improve fraction unbound, solubility and reduce lipophilicity. Therefore, **14c** ONO-TR-772 (VU60108042) stands as a valuable rodent *in vivo* tool compound to explore selective inhibition of TREK-1 and TREK-2.

**Figure 5:**
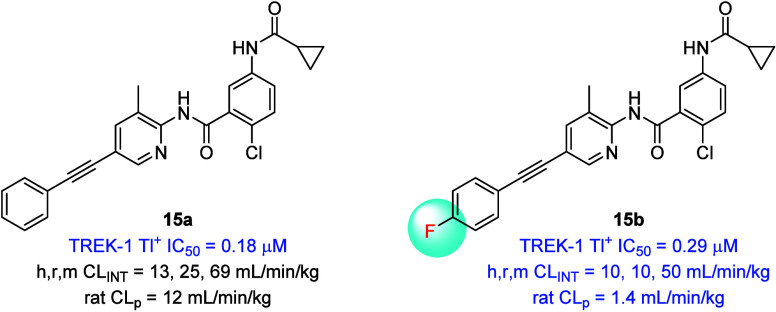
Cyclopropyl amides are viable replacements for the *N*-Boc moiety. Introduction of the 4-F phenyl moiety reduces *in vivo* rat clearance almost 10-fold.

In summary, we have developed a potent, selective, and CNS penetrant TREK inhibitor **14c** (ONO-TR-772, VU6018042) that provided proof of concept for pro-cognitive efficacy in an MK-801 challenge NOR paradigm with an MED of 10 mg/kg IP. While a valuable *in vivo* probe for the community, further efforts are ongoing and will be reported in due course.

## Supplementary Material



## Notes

Additional experimental details, methods for the synthesis and characterization of all compounds (^1^H NMR, ^13^C NMR, 2-D NMR, HRMS), *in vitro* and *in vivo* DMPK protocols and supplemental figures (PDF)

## Abbreviations

TREK: TWIK RElated K^+^ channels
MED: minimum effective dose
PK: pharmacokinetics
PBL: plasma:brain level study
NOR: novel object recognition
