# Supplementary material for: Discovery of ONO-TR-772 (VU6018042): A Highly Selective and CNS Penetrant TREK Inhibitor in Vivo Tool Compound
Source: ACS Med Chem Lett. 2025 Apr 28;16(5):896–901. doi: 10.1021/acsmedchemlett.5c00215 (PMC12067145; doi:10.1021/acsmedchemlett.5c00215)
Supplement: Supplementary file 1 [file ml5c00215_si_001.pdf]

Discovery of ONO-TR-772 (VU6018042): A highly selective and CNS penetrant TREK inhibitor *in vivo* tool compound

Motoyuki Tanaka,[1] Takahiro Mori,[2] Gakuji Hashimoto,[2] Katsukuni Mitsui,[2] Akihiro Kishi,[2] Elizabeth S. Childress,[3,4] Sean R. Bollinger,[3,4] Trevor C. Chopko,[3,4] Thomas M. Bridges,[3,4] Douglas G. Stafford,[5] Zhonping Hunag,[5] Mark A. Wolf,[5] Darren W. Engers,[3,4] Jerod S. Denton,[3,4,6] Haruto Kurata,*[1] and Craig W. Lindsley*[3,4]

Affiliation:
[1]Drug Discovery Chemistry, Ono Pharmaceutical Co., Ltd, 3-1-1 Sakurai, Shimamoto, Mishima, Osaka 618-8585, Japan
[2]Research Center of Neurology, Ono Pharmaceutical Co., Ltd, 3-1-1 Sakurai, Shimamoto, Mishima, Osaka 618-8585, Japan
[3]Warren Center for Neuroscience Drug Discovery, Vanderbilt University, Nashville, TN 37232, USA
[4]Department of Pharmacology, Vanderbilt University School of Medicine, Nashville, TN 37232, USA
[5]Curia Global, Inc., 24 Corporate Circle, Albany, NY 12203, USA
[6]Department of Anesthesiology, Vanderbilt University Medical Center, Nashville, TN 37232, USA

*To whom correspondence should be addressed at craig.lindsley@vanderbilt.edu, h.kurata@ono-pharma.com

TABLE OF CONTENTS

Eurofins Lead Profiling Screen.....S2
Ion Channel Selectivity Tables.....S3
Procedures for Biological Experiments.....S4
General Methods.....S10
Synthetic Procedures and Spectra.....S12
Supplemental Figures.....S21

Table S1. Eurofins Lead Profiling Screen Data

This is a radioligand binding panel of 72 targets including GPCRs, ion channels, transporters and nuclear hormones. Biochemical assay results are presented as the percent inhibition of specific binding at a 30 µM concentration of **ONO-2920632 (VU6011887,** Compound **19b**).

**Target/Protein**	**Species**	**% Inhibition at 10 µM**
Adenosine A_1	Human	2
Adenosine A_{2A}	Human	3
Adenosine A_3	Human	84
Adrenergic α_{1A}	Rat	40
Adrenergic α_{1B}	Rat	11
Adrenergic α_{1D}	Human	26
Adrenergic α_{2A}	Human	36
Adrenergic β_1	Human	-1
Adrenergic β_2	Human	4
Androgen (Testosterone)	Human	7
Bradykinin B_1	Human	27
Bradykinin B_2	Human	-10
Calcium Channel L-Type, Benzothiazepine	Rat	56
Calcium Channel L-Type, Dihydropyridine	Rat	76
Calcium Channel N-Type	Rat	4
Cannabinoid CB_1	Human	-23
Dopamine D_1	Human	8
Dopamine D_{2S}	Human	-1
Dopamine D_3	Human	12
Dopamine $D_{4.2}$	Human	5
Endothelin ET_A	Human	-11
Endothelin ET_B	Human	-6
Epidermal Growth Factor (EGF)	Human	-2
Estrogen ERα	Human	8
$GABA_A$, Flunitrazepam, Central	Rat	-9
$GABA_A$, Muscimol, Central	Rat	2
$GABA_{B1A}$	Human	0
Glucocorticoid	Human	-4
Glutamate, Kainate	Rat	3
Glutamate, NMDA, Agonism	Rat	2
Glutamate, NMDA, Glycine	Rat	-6
Glutamate, NMDA, Phencyclidine	Rat	-15
Histamine H_1	Human	12
Histamine H_2	Human	-12

Histamine H_3	Human	4
Imidazoline I_2, Central	Rat	10
Interleukin IL-1 R1	Human	-1
Leukotriene, Cysteinyl $CysLT_1$	Human	-7
Melatonin MT_1	Human	29
Muscarinic M_1	Human	4
Muscarinic M_2	Human	0
Muscarinic M_3	Human	7
Neuropeptide Y Y_1	Human	15
Neuropeptide Y Y_2	Human	6
Nicotinic Acetylcholine	Human	-10
Nicotinic Acetylcholine α1, Bungarotoxin	Human	-4
Opiate δ_1 (OP1, DOP)	Human	-8
Opiate κ (OP2, KOP)	Human	9
Opiate μ (OP3, MOP)	Human	15
Phorbol Ester	Mouse	-7
Platelet Activating Factor (PAF)	Human	26
Potassium Channel [K_{ATP}]	Hamster	-6
Potassium Channel hERG	Human	39
Prostanoid EP_4	Human	19
Purinergic P2X	Rat	1
Purinergic P2Y	Rat	1
Rolipram	Rat	5
Serotonin (5-HT_{1A})	Human	4
Serotonin (5-HT_{2B})	Human	31
Serotonin (5-HT_3)	Human	-4
Sigma σ_1	Human	30
Sodium Channel, Site 2	Rat	84
Tachykinin NK_1	Human	13
Thyroid Hormone	Rat	3
Transporter, Dopamine (DAT)	Human	23
Transporter, GABA	Rat	19
Transporter, Norepinephrine (NET)	Human	29
Transporter, Serotonin (SERT)	Human	-3

Cat #	Assay Name	Species
200720	Adenosine A_3	hum
214510	Calcium Channel L-Type, Benzothiazepine	rat
214600	Calcium Channel L-Type, Dihydropyridine	rat
279510	Sodium Channel, Site 2	rat

Conc.	% Inh.	IC_{50}*	K_i	n_H
1 μM	67	0.52 μM	0.48 μM	0.94
10 μM	65	2.94 μM	2.61 μM	0.52
1 μM	63	0.84 μM	0.54 μM	0.64
1 μM	61	0.71 μM	0.65 μM	1.40

Table S2. K_2P Channel selectivity

	TREK-1	TREK-2	TASK-3	TRESK	TWIK-2	TRAAK	TASK-1	TASK-2
Qpatch IC50 (uM)	0.15	0.17	>10 (19% at 10uM)	>10 (26% at 10uM)	>10 (13% at 10uM)			
MPC %inhibition at 10uM						-41% (activation)	17%	7%

Table S3. Cardiac ion channel MPC selectivity panel

MPC %inhibition at 10 uM								
Cav1.2	HCN4	Kir2.1	Kir2.2	Kv1.5	Kv4.3 /KChIP	KCNQ1/E1	Nav1.5	hERG
-0.9	0.6	-1.1	51.6 (transient)	2.4	2.1	47.3	1.1	38.4

Procedures for Biological Experiments

TREK-1 and TREK-2 thallium flux assay protocol

CHO cells stably expressing human TREK-1 (hTREK-1/CHO) were constructed at Evotec. HEK293 cells stably expressing human TREK-2 (hTREK-2/HEK) were constructed at Ono. hTREK-1/CHO or hTREK-2/HEK were plated in 384-well plates at a density of 1.0×10^4 cells/well or 2.0×10^4 cells/well, respectively, and cultured overnight. After the wash by HBSS + 20 mM HEPES buffer, cells were loaded with Thallos dye for 1 hour at room temperature. After the wash by HBSS + 20 mM HEPES buffer, test compounds, control compound (BL-1249) or 0.3% DMSO dissolved with HBSS + 20 mM HEPES buffer were added to each well. After 10 minutes incubation at room temperature, thallium stimulus buffer were added to each well and the fluorescent intensity are measured by Panoptic. The final thallium concentration was 0.36 mM in TREK-1 or 0.24 mM in TREK-2, respectively. The change of fluorescent intensity (ΔRatio) and % activation to compare the efficacy and potency of test compounds were calculated using the following equations.

Δ Ratio = (fluorescent intensity at 25 seconds after thallium addition)/(average of fluorescent intensity at pre-read)

% activation = (ΔRatio of test compound - ΔRatio of 0.3% DMSO)/(ΔRatio of 10 μM control compound - ΔRatio of 0.3% DMSO)

hERG thallium flux assay protocol

HEK293 cells stably expressing human ERG (hERG/HEK) were kindly provided by Dr. Weaver, Vanderbilt University Medical Center. hERG/HEK were plated in 384-well plates at a density of 2.0×10^4 cells/well, and cultured overnight. After the wash by HBSS + 20 mM HEPES buffer, cells were loaded with Thallos dye for 1 hour at room temperature. After the wash by HBSS + 20 mM HEPES buffer, test compounds, control compounds (dofetilide) or 0.3% DMSO dissolved with 90 mM K+ buffer was added to each well. The final K+ concentration was 45 mM. After 10 minutes incubation at room temperature, thallium stimulus buffer was added to each well and the fluorescent intensity measured by Panoptic. The final thallium concentration was 0.96 mM. The change of fluorescent intensity (ΔRatio) and % inhibition to compare the efficacy and potency of test compounds were calculated using the following equations.

Δ Ratio = (fluorescent intensity at 25 seconds after thallium addition)/(average of fluorescent intensity at pre-read)

% inhibition = 100 - (ΔRatio of test compound - ΔRatio of 1 μM control compound)/(ΔRatio of 0.3% DMSO - ΔRatio of 1 μM control compound)x100

Drug Metabolism Methods:

***In vitro* Plasma protein binding and Brain homogenate binding:** Determination of fraction unbound (f_u) in plasma was conducted *in vitro* via equilibrium dialysis using HTDialysis (HTD) membrane plates. The top half of the plate was filled with 100 uL of Dubelco's Phosphate Buffered Saline, pH 7.4 (DPBS). Compounds were diluted into plasma from each species (5 μM final concentration), which was aliquoted in triplicate to the 'bottom half' of the prepared HTD plate wells. The HTD plate was sealed and incubated for 6 hours at 37 °C. Following incubation, each well (both top and bottom halves) were transferred (20 μL) to the corresponding wells of a 96-shallow-well (V-bottom) plate. The daughter plates were then matrix-matched (DPBS side wells received equal volume of plasma, and plasma side wells received equal volume of DPBS), and extraction solution (120 μL; acetonitrile containing 50 nM carbamazepine as IS) was added to all wells of both daughter plates to precipitate protein and extract test article. The plates were then sealed and centrifuged (3500 rcf) for 10 minutes at ambient temperature. Supernatant (60 μL) from each well of the daughter plates was then transferred to the corresponding wells of new daughter plates (96-shallow-well, V bottom) containing water (Milli-Q, 60 μL/well), and the plates were sealed in preparation for LC-MS/MS analysis (see LC-MS/MS analysis method below).

The unbound fraction (f_u) was calculated following the equation below, and mean values for each species were calculated from 3 replicates. A similar approach was used to determine the degree of brain homogenate binding, which employed the same methodology and procedure with the following modifications: 1) a final compound concentration of 1 μM was used, 2) naïve rat brains were homogenized in DPBS (1:3 composition of brain: DPBS, w/w) using a Mini-Bead Beater™ machine in order to obtain brain homogenate, which was then treated in the same manner as the plasma samples in the previously described plasma protein binding assay. Fraction unbound for both plasma and brain samples was determined using Equation 4.

$$f_u = \frac{Conc_{buffer}}{Conc_{plasma}}$$

Equation 4 Determination of fraction unbound in plasma.

The diluted fraction unbound (f_u2) in brain was calculated in the same manner by using brain homogenate rather than plasma. Undiluted fraction unbound for the brain was calculated using Equation 5

$$f_u = \frac{1/4}{\left\{\left(\frac{1}{fu2}\right) - 1\right\} + 1/4}$$

Equation 5 Determination of fraction unbound in brain. F_u2 represents the diluted fraction unbound.

Intrinsic clearance: Human or rat hepatic microsomes (0.5 mg/mL) and 1 µM test compound were incubated in 100 mM potassium phosphate pH 7.4 buffer with 3 mM $MgCl_2$ at 37 °C with constant shaking. After a 5 min preincubation, the reaction was initiated by the addition of NADPH (1 mM). At selected time intervals between 0 ~ 60 min (e.g. 0, 3, 7, 15, 25, 45 and/or 60 min), aliquots were taken and subsequently placed into a 96-well plate containing cold acetonitrile with an internal standard (50 ng/mL carbamazepine). Plates were then centrifuged at 3000 rcf (4 °C) for 10 min, and the supernatant was transferred to a separate 96-well plate and diluted 1:1 with water for LC/MS/MS analysis. The *in vitro* half-life ($T_{1/2}$, min, Eq. 1), intrinsic clearance (CL_{int}, mL/min/kg, Eq. 2), and subsequent predicted hepatic clearance (CL_{hep}, mL/min/kg, Eq. 3) were determined employing the following equations:

(1) $T_{1/2} = \frac{Ln(2)}{K}$

where k represents the slope from linear regression analysis of the natural log percent remaining of a test compound as a function of incubation time

(2) $CL_{int} = \frac{0.693}{in\ vitro T_{1/2}}\ x\ \frac{mL\ incubation}{mg\ microsomes}\ x\ \frac{45\ mg\ microsomes}{gram\ liver}\ x\ \frac{20^a\ gram\ liver}{kg\ body\ wt}$

[a]scale-up factors: of 20 (human) or 45 (rat) or 87.5 (mouse)

(3) $CL_{hep} = \frac{Q_h \cdot CL_{int}}{Q_h + CL_{int}}$

where Q_h (hepatic blood flow, mL/min/kg) is 21 (human) or 70 (rat) or 90 (mouse).

LC/MS/MS Bioanalysis of Samples from Plasma Protein Binding and Intrinsic Clearance Assays:

Samples were analyzed on a Thermo Electron TSQ Quantum Ultra triple quad mass spectrometer (San Jose, CA) via electrospray ionization (ESI) with two Themo Electron Accella pumps (San Jose, CA), and a Leap Technologies CTC PAL autosampler (Carrboro, NC). Analytes were separated by gradient elution on a dual column system with two Thermo Hypersil Gold (2.1 x 30 mm, 1.9 µm) columns (San Jose, CA) thermostated at 40 °C. HPLC mobile phase A was 0.1% formic acid in water and mobile phase B was 0.1% formic acid in acetonitrile. The gradient started at 10% B after a 0.2 min hold and was linearly increased to 95% B over 0.8 min; hold at 95% B for 0.2 min; returned to 10% B in 0.1 min. The total run time was 1.3 min and the HPLC flow rate was 0.8 mL/min. While pump 1 ran the gradient method, pump 2 equilibrated the alternate column isocratically at 10% B. Compound optimization, data collection, and processing were performed using Thermo Electron's QuickQuan software (v2.3) and Xcalibur (v2.0.7 SP1).

***In vivo* DMPK experimental:**

Determination of brain to plasma ratio:

Animal care and use

All animal study procedures were approved by the Institutional Animal Care and Use Committee and were conducted in accordance with the National Institutes of Health regulations of animal care covered in Principles of Laboratory Animal Care (National Institutes of Health). All rats were fasted overnight prior to testing.

In-life phase

For determination of the brain over plasma ratio (K_p), compounds were formulated in 8% ethanol, 32% PEG400 and 60% DMSO (*v/v/v*) and administered as a single 0.2 mg/kg IV dose (1 mL/kg) to male, Sprague Dawley rats (n = 1) via injection into a surgically-implanted jugular vein catheter. At 15 min post dosing, blood sample was collected into chilled, K_2EDTA anticoagulant-

fortified tube and immediately placed on wet ice. The blood sample was then centrifuged (1700 rcf, 5 minutes, 4 °C) to obtain plasma sample. At the same post-administration time point, a whole brain sample was obtained by rapid dissection, rinsed with PBS, and immediately frozen in individual tissue collection box (dry ice). All brain and plasma samples were stored at -80 °C until analysis by LC-MS/MS.

Sample Analysis: Concentrations in plasma and brain homogenates were quantified by liquid chromatography tandem mass spectrometry (LC-MS/MS). Whole brains were homogenized in 3 mL of 70:30 IPA:water in a mini bead beater for 3 min, and centrifuged at 3,500 g for 5 min. 5 uL of the supernatant was diluted in 15 uL of blank plasma for quantification of the analytes. Plasma samples were centrifuged at 3,500 g for 5 min. A standard curve was generated by diluting the analytes DMSO stocks with blank plasma to obtain a final concentration of 10,000 ng/mL followed by a serial dilution down to 0.5 ng/mL. Quality controls were generated by a serial dilution of the 5,000 ng/mL standard curve solution in blank plasma to obtain 3 concentrations of 500, 50, and 5 ng/mL. 20 uL of brain diluted in plasma, plasma, blank plasma, standard curve and QC samples were loaded in a V-bottom 96-well plate. 120 uL of acetonitrile containing 0.05 uM carbamazepine (internal standard) was added to each well and the plate was centrifuged at 3,500 g for 5 min. 60 uL of the supernatant of each well (protein free) was transferred to a new 96-well plate containing 60 uL of water. The plates were sealed for analysis by LC-MS/MS.

Plasma and brain tissue samples originating from *in vivo* studies were analyzed by electrospray ionization using an AB Sciex Q-TRAP 5500 (Foster City, CA) that was coupled to a Shimadzu LC-20AD pump (Columbia, MD) and a Leap Technologies CTC PAL auto-sampler (Carrboro, NC). Analytes were separated by gradient elution using a C18 column (3 x 50 mm, 3 mm; Fortis Technologies Ltd, Cheshire, UK) that was thermostated at 40 °C. HPLC mobile phase A was 0.1% formic acid in water (pH unadjusted); mobile phase B was 0.1% formic acid in acetonitrile (pH unadjusted). A 10% B gradient was held for 0.2 min and was linearly increased to 90% B over 0.8 min, with an isocratic hold for 0.5 min, before transitioning to 10% B over 0.05 min. The column was re-equilibrated (1 min) before the next sample injection. The total run time was 2.55 min, and the HPLC flow rate was 0.5 mL/min. The source temperature was set at 500 °C, and mass spectral analyses were performed using a Turbo-Ion spray source in positive ionization mode (5.0-kV spray voltage) and using multiple-reaction monitoring of transitions specific for the analytes. All data were analyzed using AB Sciex Analyst 1.5.1 software.

Brain plasma concentration ratio (K_p) was calculated by dividing brain concentration by plasma concentration for each animal. Unbound brain to unbound plasma concentration ratio ($K_{p,uu}$) is calculated using the following formula: $K_{p,uu}$ = (Brain ng/g x brain fu)/(plasma ng/mL x plasma fu).

For determination of the plasma concentration-time profile, compounds were formulated in methyl cellulose and administered as a 3 mg/kg PO dose to male C57BL/6 mouse or SD rat (*n* = 2). For the same determination, compounds were formulated in HP-β-CD and administered as a 0.1 mg/kg IV dose to male beagle dog (*n* = 2).

Plasma samples originating from *in vivo* studies were analyzed by electrospray ionization using a Q-TRAP 5500 (AB Sciex) that was coupled to a Prominence UFLC XR (Shimadzu Corporation). Analytes were separated by gradient elution using a Shim-pack XR-ODS II (2 x 75 mm, 2.2μm, Shimadzu Corporation) that was thermostated at 40 °C. HPLC mobile phase A was 5 mM ammonium acetate containing 0.2% formic acid in water (pH unadjusted); mobile phase B was acetonitrile. A 10% B gradient was linearly increased to 90% B over 1.5 min, with an isocratic hold for 1.5 min, before transitioning to 10% B over 0.1 min. The column was re-equilibrated (0.9 min) before the next sample injection. The total run time was 4.0 min, and the HPLC flow rate was 0.5 ml/min. The source temperature was set at 600 °C, and mass spectral analyses were performed using a Turbo-Ion spray source in positive ionization mode and using multiple-reaction monitoring of transitions specific for the analytes. All data were analyzed using AB Sciex Analyst software.

Experimental for the MK-801 NOR assay.

A plastic chamber (W 345×L 400×H 180 mm) was used for the novel object recognition test. The light intensity in the test room was maintained at approximately 20 lux. The novel object recognition test was conducted over two days. On the first day, mice were placed in the chamber without any objects and allowed to explore freely for 10 minutes to acclimate to the experimental environment. On the second day, TR-772 (3, 10, 30 mg/kg), clozapine (1 mg/kg), or a vehicle was administered intraperitoneally, and 3 hours and 30 minutes later, MK-801 (0.2 mg/kg) was administered intraperitoneally. Thirty minutes after the MK-801 challenge, the acquisition trial

was conducted by placing two identical objects 10 cm away from the walls in the chamber, and the mice were allowed to explore freely for 10 minutes. Ninety minutes after the acquisition trial, the retention trial was conducted by placing one familiar object (identical to the objects used in the acquisition trial) and one novel object in the chamber, and the mice were allowed to explore freely for 10 minutes. The retention trial was recorded with a video camera placed above the chamber, and the exploration time for each object during the 10-minute trial was measured. An object was considered to be explored if the mouse's nose was within 1 cm of the object. After video analysis, the discrimination index (%) was calculated using the following formula:

Discrimination index (%) = (Novel – Familiar) / Total exploring time × 100

General Methods

All NMR spectra were recorded on a 400 MHz AMX Bruker NMR spectrometer or a 600 MHz VNS600 Agilent NMR spectrometer or Bruker 500 MHz Asend/Avance III HP NMR spectrometer. ^{1}H and ^{13}C chemical shifts are reported in δ values in ppm downfield with the deuterated solvent as the internal standard. Data are reported as follows: chemical shift, multiplicity (s = singlet, d = doublet, t = triplet, q = quartet, br = broad, m = multiplet), integration, coupling constant (Hz). Low resolution mass spectra were obtained on an Agilent 6120/6150 or Waters QDa (Performance) SQ MS with ESI source. *Method A (Agilent 6120/6150):* MS parameters were as follows: fragmentor: 70, capillary voltage: 3000 V, nebulizer pressure: 30 psig, drying gas flow: 13 L/min, drying gas temperature: 350 °C. Samples were introduced via an Agilent 1290 UHPLC comprised of a G4220A binary pump, G4226A ALS, G1316C TCC, and G4212A DAD with ULD flow cell. UV absorption was generally observed at 215 nm and 254 nm with a 4 nm bandwidth. Column: Waters Acquity BEH C18, 1.0 x 50 mm, 1.7 um. Gradient conditions: 5% to 95% CH_3CN in H_2O (0.1% TFA) over 1.4 min, hold at 95% CH_3CN for 0.1 min, 0.5 mL/min, 55 °C. *Method B (Agilent 6120/6150):* MS parameters were as follows: fragmentor: 100, capillary voltage: 3000 V, nebulizer pressure: 40 psig, drying gas flow: 11 L/min, drying gas temperature: 350 °C. Samples were introduced via an Agilent 1200 HPLC comprised of a degasser, G1312A binary pump, G1367B HP-ALS, G1316A TCC, G1315D DAD, and a Varian

380 ELSD (if applicable). UV absorption was generally observed at 215 nm and 254 nm with a 4 nm bandwidth. Column: Thermo Accucore C18, 2.1 x 30 mm, 2.6 um. Gradient conditions: 7% to 95% CH_3CN in H_2O (0.1% TFA) over 1.6 min, hold at 95% CH_3CN for 0.35 min, 1.5 mL/min, 45 °C. *Method C (Waters QDa (Performance) SQ MS)*: MS parameters were as follows: cone voltage: 15 V, capillary voltage: 0.8 kV, probe temperature: 600 °C. Samples were introduced via an Acquity I-Class PLUS UPLC comprised of a BSM, FL-SM, CH-A, and PDA. UV absorption was generally observed at 215 nm and 254 nm; 4 nm bandwidth. Column: Phenomenex EVO C18, 1.0 x 50 mm, 1.7 um. Column temperature: 55 °C. Flow rate: 0.4 mL/min. Default gradient: 5% to 95% CH_3CN (0.05% TFA) in H_2O (0.05% TFA) over 1.4 min (curve 6), hold at 95% CH_3CN for 0.1 min. “Polar” (2% to 70% CH_3CN (0.05% TFA) in H_2O (0.05% TFA) over 0.8 min (curve 6), transition to 95% CH_3CN over 0.1 min (curve 6), hold at 95% CH_3CN for 0.6 min.) and “Non-Polar” (40% to 95% CH_3CN (0.05% TFA) in H_2O (0.05% TFA) over 1.4 min (curve 6), hold at 95% CH_3CN for 0.1 min.) gradients were also available. *Method D (Waters QDa (Performance) SQ MS):* MS parameters were as follows: cone voltage: 15 V, capillary voltage: 0.8 kV, probe temperature: 600 °C. Samples were introduced via an Acquity I-Class PLUS UPLC comprised of a BSM, FL-SM, CH-A, and PDA. UV absorption was generally observed at 215 nm and 254 nm with a 4 nm bandwidth. Column: Phenomenex EVO C18, 1.0 x 50 mm, 1.7 um. Column temperature: 55° C. Flow rate: 0.4 mL/min. Default gradient: 5% to 95% CH_3CN in H_2O (5 mM NH_4HCO_3) over 1.4 min (curve 6), hold at 95% CH_3CN for 0.1 min. “Polar” (2% to 70% CH_3CN in H_2O (5 mM NH_4HCO_3) over 0.8 min (curve 6), transition to 95% CH_3CN over 0.1 min (curve 6), hold at 95% CH_3CN for 0.6 min.) and “Non-Polar” (40% to 95% CH_3CN in H_2O (5 mM NH_4HCO_3) over 1.4 min (curve 6), hold at 95% CH_3CN for 0.1 min.) gradients were also available. *Method E*: Reversed-phase LCMS analysis was obtained on a SHIMADZU LCMS-2020 with ESI source. MS parameters were as follows: Mobile Phase: 0.1% TFA in water (solvent A) and 0.1% TFA in acetonitrile (solvent B), using the elution holding at 5% (solvent B) for 0.1 minutes, gradient 5%-95% (solvent B) over 1.1 minutes and holding at 95% for 0.4 minutes at a flow rate of 1.0 mL/min; Column: YMC Triart C18 Φ2.0 mm* L30mm; Wavelength: UV 220nm, 254nm; Column temperature: 30 °C; detector MS, ELSD; MS ionization: ESI. High resolution mass spectra were obtained on a Thermo Fisher Scientific LTQ Orbitrap XL (*Method (i)*) or a Waters Corporation SYNAPT G2-Si (*Method (ii)*) with ESI source. MS parameters were as follows: *Method (i)*: Capillary Temp: 340 °C, Sheath Gas Flow: 55, Aux Gas

Flow: 15, Positive Polarity Source Voltage: 4500 V, Source Current: 100 μA, Capillary Voltage: 30 V, Tube Lens: 70 V. Samples were introduced via an Shimadzu UFLCXR HPLC. UV absorption was observed at DAD (190-400 nm). Column: Imtakt Unison UK-C18, 3 μm, 2 x 100 mm. Gradient conditions: 10% to 90% CH_3CN (0.1% formic acid) over 10 min, 0.3 mL/min, 40 ºC. *Method (ii)*: Capillary: 2.0 kV, Sampling Cone: 10, Source Offset: 50, Source: 120 ºC, Desolvation: 400 ºC, Gas Flow Cone Gas: 50 L/h, Desolvation Gas: 1000 L/h, Nebuliser Gas: 6.5 Bar. Samples were introduced via an Waters ACQUITY UPLC I-Class. UV absorption was observed at DAD (210-400nm). Column: YMC-Triart C18, S-1.9 um, 12 nm, 30 x 2.0 mm I.D. Gradient conditions: 5% to 95% CH_3CN (0.1% formic acid) over 10 min, 0.3 mL/min, 40 ºC. For compounds that were purified on a Gilson preparative reversed-phase HPLC, the system comprised of a 333 aqueous pump with solvent selection valve, 334 organic pump, GX 271 or GX-281 liquid hander, two column switching valves, and a 155 UV detector. UV wavelength for fraction collection was user-defined, with absorbance at 254 nm always monitored. Method 1: Phenomenex Axia-packed Luna C18, 30 x 50 mm, 5 μm column. Mobile phase: CH_3CN in H_2O (0.1% TFA). Gradient conditions: 0.75 min equilibration, followed by user defined gradient (starting organic percentage, ending organic percentage, duration), hold at 95% CH_3CN in H_2O (0.1% TFA) for 1 min, 50 mL/min, 23 ºC. Method 2: Phenomenex Axia packed Gemini C18, 50 x 250 mm, 10 um column. Mobile phase: CH_3CN in H_2O (0.1% TFA). Gradient conditions: 7 min equilibration, followed by user defined gradient (starting organic percentage, ending organic percentage, duration), hold at 95% CH_3CN in H_2O (0.1% TFA) for 7 min, 120 mL/min, 23 ºC. Solvents for extraction, washing and chromatography were HPLC grade. All compounds are >95% purity by HPLC.

Chemical Synthesis.

Compound 6 (ONO-0606822)

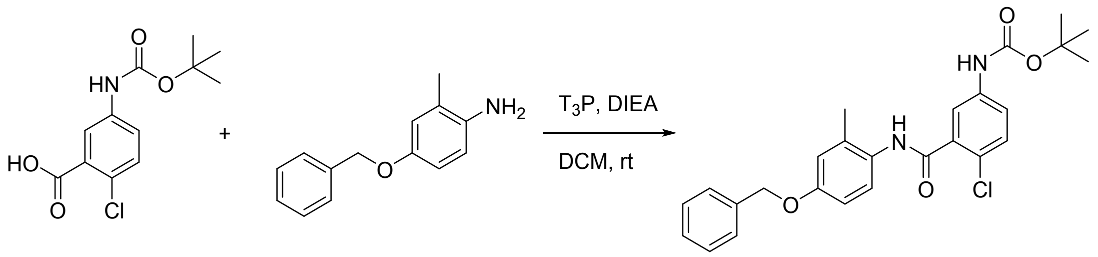

6

***tert*-Butyl (3-((4-(benzyloxy)-2-methylphenyl)carbamoyl)-4-chlorophenyl)carbamate (6):** To a solution of 5-(*tert*-butoxycarbonylamino)-2-chloro-benzoic acid (500 mg, 1.84 mmol), 4-(benzyloxy)-2-methylaniline (393 mg, 1.84 mmol), DIEA (0.96 mL, 5.5 mmol) in DCM (30 mL) was added a solution of propylphosphonic anhydride (T_3P) in ethyl acetate (50%, 1.6 mL, 2.7 mmol). After the reaction mixture was stirred for 32 h at room temperature, the reaction mixture was poured into NH_4Cl-aq and extracted with DCM twice. The combined organic layer was washed with brine and dried over $MgSO_4$. The filtrate was concentrated under reduced pressure to give a crude product, which was purified by column chromatography on silica gel and the concentrated residue was triturated with methanol to yield **6** as a white powder (660 mg, 77% yield). m/z $[M+1]^+$: 467.4; LCMS retention time: 1.25 min (LCMS Method E); ^1H NMR (400 MHz, DMSO-d_6) δ 9.80 (br s, 1H), 9.66 (br s, 1H), 7.72 (d, J = 2.5 Hz, 1H), 7.51 (dd, J = 9.0, 2.5 Hz, 1H), 7.47 – 7.39 (m, 5H), 7.36 – 7.32 (m, 1H), 7.24 (d, J = 9.0 Hz, 1H), 6.94 (d, J = 2.5 Hz, 1H), 6.87 (dd, J = 9.0, 2.5 Hz, 1H), 5.11 (s, 2H), 2.25 (s, 3H), 1.49 (s, 9H).

IM- 1 for Compound 10a

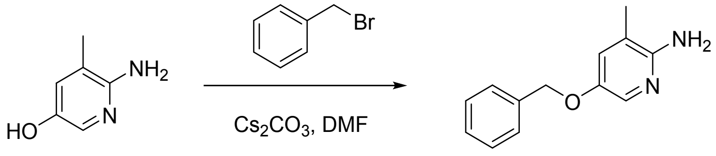

7 **IM-1**

5-(Benzyloxy)-3-methylpyridin-2-amine (IM-1): A dry 50 mL round bottom flask, equipped with a magnetic stir bar and nitrogen atmosphere, was charged with 6-amino-5-methylpyridin-3-ol (compound **7**, 90 mg, 0.73 mmol) and cesium carbonate (360 mg, 1.11 mmol) in *N,N*-dimethylformamide (6 mL). Benzyl bromide (0.086 mL, 0.73 mmol) was added and the reaction mixture stirred at ambient temperature for 22 h. After this time, the reaction mixture was diluted with water (40 mL) and extracted with ethyl acetate (3 × 30 mL). The combined organic layers were washed with brine (20 mL) and concentrated under reduced pressure. The resulting residue was purified by chromatography (silica, heptane to ethyl acetate) to afford **IM-1** (18 mg, 12%) as an off-white solid: ^1H NMR (500 MHz, $CDCl_3$) δ 7.72 (d, J = 2.5 Hz, 1H), 7.42–7.36 (m, 4H), 7.33–7.30 (m, 1H), 7.03 (d, J = 2.5 Hz, 1H), 5.00 (s, 2H), 4.12 (br s, 2H), 2.12 (s, 3H).

Compound 10a

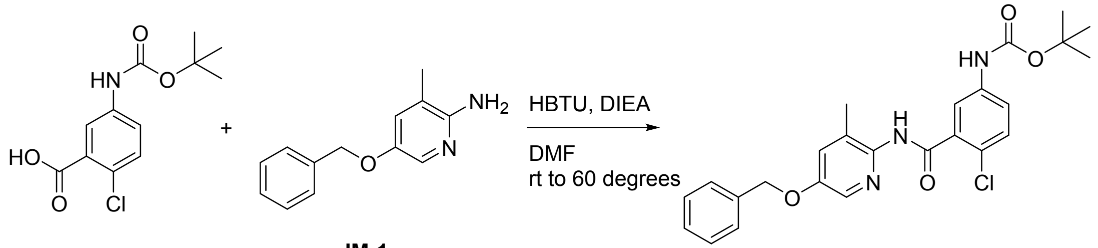

IM-1

10a

***tert*-Butyl (3-((5-(benzyloxy)-3-methylpyridin-2-yl)carbamoyl)-4-chlorophenyl)carbamate** (**10a**): A dry 50 mL round bottom flask, equipped with a magnetic stir bar and nitrogen atmosphere, was charged with 5-((*tert*-butoxycarbonyl)amino)-2-chlorobenzoic acid (26 mg, 0.096 mmol), and *N,N*-diisopropylethylamine (0.050 mL, 0.29 mmol) in *N,N*-dimethylformamide (0.75 mL). o-benzotriazol-1-yl-tetramethyluronium hexafluorophosphate (HBTU, 45 mg, 0.12 mmol) was added and the reaction mixture stirred at ambient temperature for 15 minutes. A solution of 5-(benzyloxy)-3-methylpyridin-2-amine (**IM-1**, 25 mg, 0.12 mmol) in *N,N*-dimethylformamide (1 mL) was added and the reaction mixture heated at 60 °C for 20 h. After this time, the reaction mixture was cooled to room temperature, diluted with water (30 mL) and extracted with ethyl acetate (3 × 20 mL). The combined organic layers were washed with brine (20 mL), dried over sodium sulfate, filtered and the filtrate concentrated under reduced pressure. The resulting residue was purified by chromatography (silica, heptane to ethyl acetate) to afford **10a** (8 mg, 18%) as a white solid. m/z $[M+1]^+$: 468.4; LCMS retention time: 1.15 min (LCMS Method E); ^1H NMR (500 MHz, $CDCl_3$) δ 8.16 (br s, 1H), 8.04 (s, 1H), 7.65–7.62 (m, 2H), 7.44–7.39 (m, 4H), 7.37–7.33 (m, 2H), 7.22 (d, J = 3.0 Hz, 1H), 6.56 (s, 1H), 5.11 (s, 2H), 2.36 (s, 3H), 1.52 (s, 9H).

IM-2 for Compound 10b

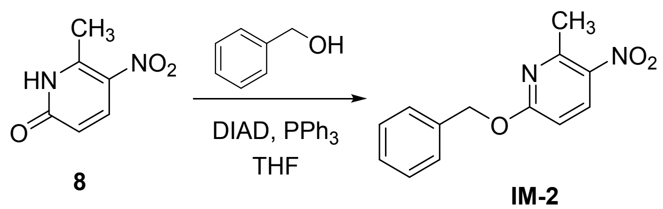

8

IM-2

6-(Benzyloxy)-2-methyl-3-nitropyridine (IM-2): A dry 50 mL round bottom flask, equipped with a magnetic stir bar and nitrogen atmosphere, was charged with 6-methyl-5-nitropyridin-

2(1*H*)-one (compound **8**, 260 mg, 1.69 mmol), benzyl alcohol (177 mg, 1.64 mmol) and triphenylphosphine (878 mg, 3.35 mmol) in tetrahydrofuran (3 mL) and the reaction mixture cooled to 0 °C. Diisopropyl azodicarboxylate (0.80 mL, 4.1 mmol) was added and the reaction mixture slowly warmed to ambient temperature while stirring overnight. After this time, the reaction mixture was concentrated under reduced pressure and the resulting residue purified by chromatography (silica, heptane to 30% ethyl acetate/heptane), followed by chromatography (silica, heptane to 25% ethyl acetate/heptane) to afford **IM-2** (95.5 mg, 24%) as an off-white solid. m/z $[M+1]^+$: 245; ^{1}H NMR (500 MHz, DMSO-d_6) δ 8.39 (d, J = 9.0 Hz, 1H), 7.50-7.47 (m, 2H), 7.42-7.38 (m, 2H), 7.36-7.33 (m, 1H), 6.94 (d, J = 9.0 Hz, 1H), 5.46 (s, 2H), 2.74 (s, 3H).

IM-3 for Compound 10b

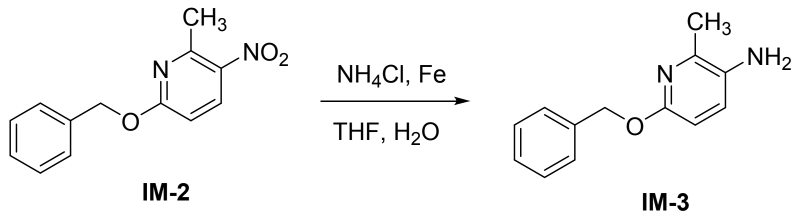

6-(Benzyloxy)-2-methylpyridin-3-amine (IM-3): A dry 10 mL microwave vial, equipped with a magnetic stir bar and nitrogen atmosphere, was charged with 6-(benzyloxy)-2-methyl-3-nitropyridine (**IM-2**, 42.8 mg, 0.175 mmol) in tetrahydrofuran (3 mL). Ammonium chloride (40 mg, 0.75 mmol) in water (2 mL) was added and the reaction mixture stirred at 75 °C for 30 minutes. The reaction mixture was cooled, iron powder (39 mg, 0.70 mmol) was added and the reaction mixture stirred at 75 °C for 5 h. After this time, the reaction mixture was cooled to room temperature and filtered through a pad of diatomaceous earth. The filtrate was neutralized with saturated aqueous sodium bicarbonate (40 mL) and extracted with ethyl acetate (3 × 10 mL). The combined organic layers were washed with brine (20 mL), dried over sodium sulfate, filtered and the filtrate concentrated under reduced pressure to afford **IM-3** (32.5 mg, 87%) as a yellow oil. m/z $[M+1]^+$: 215; ^{1}H NMR (500 MHz, DMSO-d_6) δ 7.42-7.40 (m, 2H), 7.37-7.34 (m, 2H), 7.30-7.27 (m, 1H), 6.99 (d, J = 8.5 Hz, 1H), 6.46 (d, J = 8.5 Hz, 1H), 5.19 (s, 2H), 4.51 (br s, 2H), 2.20 (s, 3H).

Compound 10b

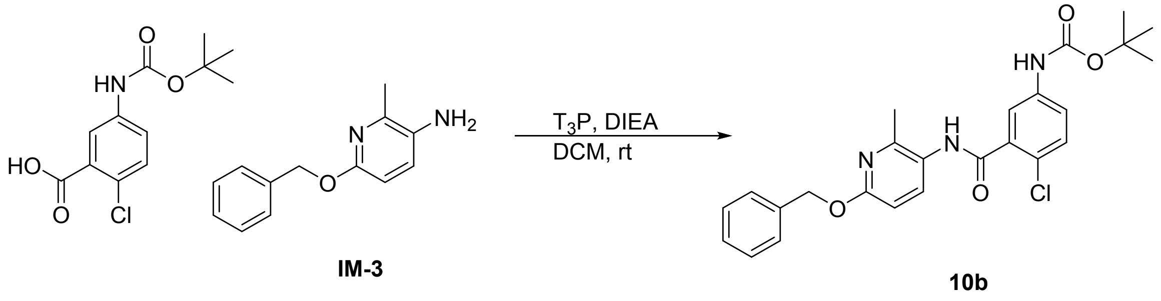

***tert*-Butyl (3-((6-(benzyloxy)-2-methylpyridin-3-yl)carbamoyl)-4-chlorophenyl)carbamate (10b)**: A dry 100 mL round bottom flask, equipped with a magnetic stir bar and nitrogen atmosphere, was charged with 6-(benzyloxy)-2-methylpyridin-3-amine (**IM-3**, 30.9 mg, 0.144 mmol), 5-((*tert*-butoxycarbonyl)amino)-2-chlorobenzoic acid (41 mg, 0.15 mmol) and *N,N*-diisopropylethylamine (0.10 mL, 0.57 mmol) in dichloromethane (15 mL). Propylphosphonic anhydride solution (50 wt% in ethyl acetate, 0.10 mL, 0.34 mmol) was added and the reaction mixture stirred at ambient temperature overnight. After this time, the reaction mixture was concentrated under reduced pressure and the resulting residue purified by chromatography (silica, heptane to 30% ethyl acetate/heptane) to afford **10b** (29.9 mg, 42%) as a white solid. m/z $[M+1]^+$: 468.3; LCMS retention time: 1.22 min (LCMS Method E); ^{1}H NMR (500 MHz, DMSO-d_6) δ 10.00 (s, 1H), 9.66 (s, 1H), 7.74 (d, J = 2.5 Hz, 1H), 7.65 (d, J = 9.0 Hz, 1H), 7.51 (dd, J = 8.5, 2.5 Hz, 1H), 7.47–7.45 (m, 2H), 7.43 (d, J = 9.0 Hz, 1H), 7.39–7.37 (m, 2H), 7.33–7.30 (m, 1H), 6.74 (d, J = 8.5 Hz, 1H), 5.34 (s, 2H), 2.39 (s, 3H), 1.48 (s, 9H). Note: Using a 1-D ^{1}H NMR experiment, excitation of the peak at 5.34 ppm resulted in an NOE observed at 6.74 ppm.

IM-4 for Compound 10c

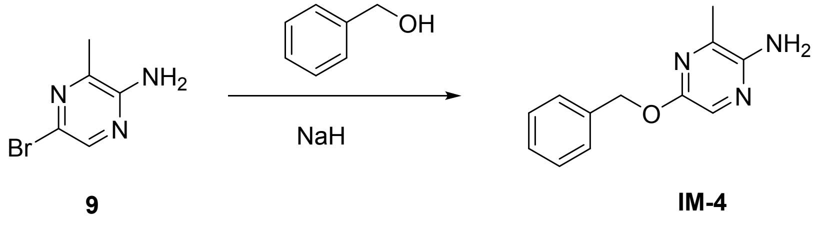

5-(Benzyloxy)-3-methylpyrazin-2-amine (IM-4): A dry 10 mL microwave vial, equipped with a magnetic stir bar and nitrogen atmosphere, was charged with 5-bromo-3-methylpyrazin-2-amine **9** (135 mg, 0.718 mmol) in benzyl alcohol (0.80 mL, 7.7 mmol) and the reaction mixture cooled

to 0 °C. 60% Sodium hydride in mineral oil (31.6 mg, 0.790 mmol) was added in two portions; one at 0 °C and the other at ambient temperature. The reaction vial was sealed and the reaction mixture heated at 120 °C for 5 h then stirred at ambient temperature overnight. After this time, the reaction mixture was diluted with water (40 mL) and extracted with dichloromethane (3 × 25 mL). The combined organic layers were washed with brine (20 mL) and concentrated under reduced pressure. The resulting residue was purified by chromatography (silica, heptane to 40% ethyl acetate/heptane), followed by reverse phase chromatography (C18, water to acetonitrile). The combined column fractions were diluted with water (20 mL) and extracted with ethyl acetate (3 × 20 mL). The combined organic layers were washed with brine (20 mL), dried over sodium sulfate, filtered, and the filtrate concentrated under reduced pressure to afford **IM-4** (52 mg, 34%) as an off-white solid. m/z $[M+1]^+$: 216; ^{1}H NMR (500 MHz, $CDCl_3$) δ 7.66–7.65 (m, 1H), 7.46–7.43 (m, 2H), 7.38–7.35 (m, 2H), 7.33–7.29 (m, 1H), 5.29 (s, 2H), 4.06 (br s, 2H), 2.35 (d, J = 1.0 Hz, 3H).

Compound 10c

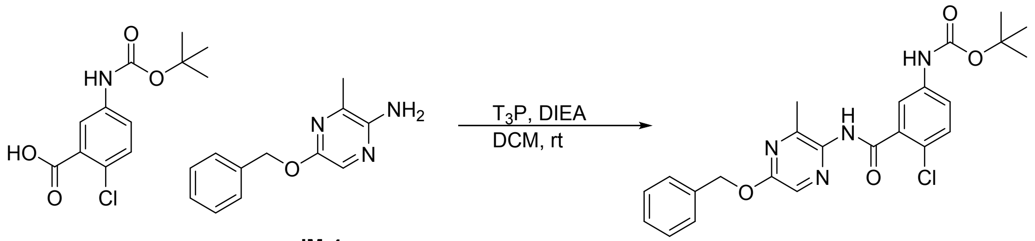

IM-4

10c

***tert*-Butyl (3-((5-(benzyloxy)-3-methylpyrazin-2-yl)carbamoyl)-4-chlorophenyl)carbamate (10c)**: A dry 250 mL round bottom flask, equipped with a magnetic stir bar and nitrogen atmosphere, was charged with 5-(benzyloxy)-3-methylpyrazin-2-amine (**IM-4**, 39 mg, 0.18 mmol), 5-((*tert*-butoxycarbonyl)amino)-2-chlorobenzoic acid (46 mg, 0.17 mmol), and *N,N*-diisopropylethylamine (0.090 mL, 0.52 mmol) in dichloromethane (3 mL). Propylphosphonic anhydride solution (50 wt% in ethyl acetate, 0.20 mL, 0.34 mmol) was added and the reaction mixture stirred at ambient temperature overnight. After this time, the reaction mixture was concentrated under reduced pressure and the resulting residue purified by chromatography (silica, heptane to 30% ethyl acetate/heptane), followed by (C18, water to acetonitrile) to afford **10c** (20.4 mg, 26%) as a white solid. m/z $[M+1]^+$: 469.3; LCMS retention time: 1.23 min (LCMS Method

E); ^{1}H NMR (500 MHz, $CDCl_3$) δ 8.27 (br s, 1H), 7.92 (s, 1H), 7.69 (br s, 1H), 7.60 (d, J = 8.0 Hz, 1H), 7.48-7.46 (m, 2H), 7.41-7.32 (m, 4H), 6.63 (s, 1H), 5.40 (s, 2H), 2.54 (s, 3H), 1.52 (s, 9H); ^{13}C NMR (125 MHz, $CDCl_3$) δ 158.15, 152.36, 146.81, 137.82, 137.55, 136.47, 134.44, 131.04, 130.27, 128.56, 128.33, 128.19, 124.21, 121.75, 120.03, 81.33, 68.34, 28.28, 21.14.

General Procedures for alkyne compounds with Boc-aniline

To a microwave vial or a round bottom flask equipped with a magnetic stir bar were added a benzoic acid (1 equiv.), an Ar-NH_2 (1.1 equiv.), DIEA or pyridine (3 equiv.) and DCM (0.3 - 0.6 mol/L), followed by PyClU (1.5 equiv.). This mixture was allowed to stir at 100 °C for 45 min under the microwave condition or 50 °C in an oil bath for 1 hour. The reaction mixture was diluted with saturated aqueous sodium bicarbonate solution and extracted with EtOAc (THF was also added in the case that a desired product was not dissolved in EtOAc). The combined organic extracts washed with water, brine and the filtrate was concentrated under a stream of air or reduced pressure. The crude residue was purified by reverse-phase HPLC (eluting with 0.1% trifluoroacetic acid and acetonitrile) or trituration with a suitable solvent(s) such as DCM / hexane to afford the amide compound with Ar-I or Ar-Br for Sonogashira coupling reaction.

To a microwave vial or a round bottom flask equipped with a magnetic stir bar were added the amide compound with Ar-I or Ar-Br (1 equiv.), copper(I) iodide (0.20 equiv.), DIEA (10 equiv.), trans-dichlorobis(triphenylphosphine)palladium(II) (0.20 equiv.) and DMF (0.1 - 0.3 mol/L), This mixture was degassed with N_2 and then diluted with an alkyne (5 equiv.). The mixture was heated to 60 °C for 2 hours under the microwave condition or 60 °C in an oil bath for 3 hours. Afterwards LCMS indicated production of the desired product. The reaction mixture was filtered through a pad of celite with $CHCl_3$:IPA (3 : 1). The crude solution was concentrated. The residue was purified on reverse-phase HPLC (eluting with 65-95% ACN in H_2O/NH_4OH) or column chromatography on silica gel and/or recrystallization with a suitable solvent(s) such as THF / ethyl acetate / hexane to afford the alkyne compounds with Boc-aniline **11**, **12**, **13a**, **14a**, **14b** and **14d**.

Compound 11

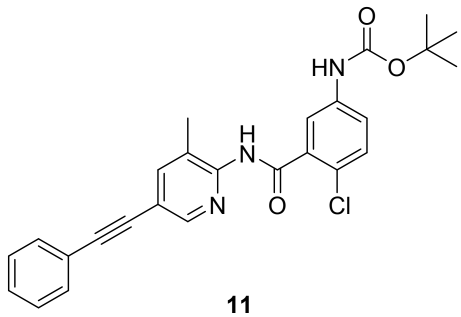

11

***tert*-Butyl (4-chloro-3-((3-methyl-5-(phenylethynyl)pyridin-2-yl)carbamoyl)phenyl)carbamate (11)**: Compound **11** was synthesized by the general procedures. m/z $[M+1]^+$: 462.4; LCMS retention time: 1.24 min (LCMS Method E); ^1H NMR (600 MHz, DMSO-d_6) δ 10.71 (s, 1H), 9.68 (s, 1H), 8.46 (s, 1H), 7.95 (s, 1H), 7.74 (s, 1H), 7.61–7.59 (m, 2H), 7.53 (d, J = 8.8 Hz, 1H), 7.47 -7.46 (m, 3H), 7.42 (d, J = 8.8 Hz, 1H), 2.32 (s, 3H), 1.49 (s, 9H).

Compound 12

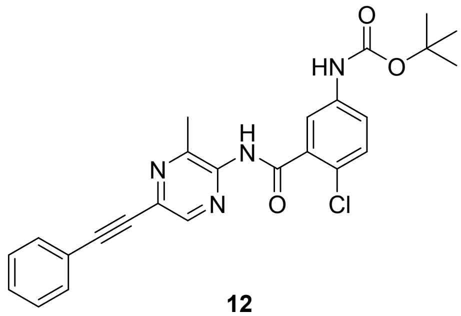

12

***tert*-Butyl (4-chloro-3-((3-methyl-5-(phenylethynyl)pyrazin-2-yl)carbamoyl)phenyl)carbamate (12)**: Compound **12** was synthesized by the general procedures. m/z $[M+1]^+$: 463.4; LCMS retention time: 1.23 min (LCMS Method E); ^1H NMR (600 MHz, DMSO-d_6) δ 11.04 (s, 1H), 9.70 (s, 1H), 8.59 (s, 1H), 7.77 (s, 1H), 7.67–7.66 (m, 2H), 7.55 -7.48 (m, 4H), 7.44 (d, J = 8.8 Hz, 1H), 2.55 (s, 3H), 1.49 (s, 9H).

Compound 13a

***tert*-Butyl (4-chloro-3-((3-fluoro-5-(phenylethynyl)pyridin-2-yl)carbamoyl)phenyl)carbamate (13a)**:

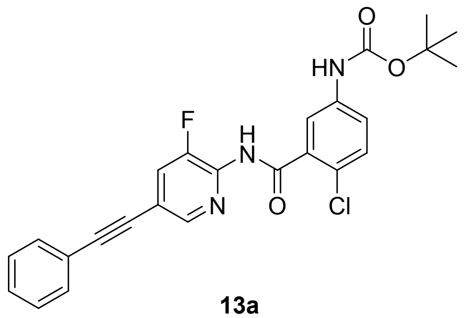

13a

Compound **13a** was synthesized by the general procedures. m/z $[M+1]^+$: 466.3; LCMS retention time: 1.26 min (LCMS Method E); ^{1}H NMR (600 MHz, DMSO-d_6) δ 11.04 (s, 1H), 9.70 (s, 1H), 8.48 (s, 1H), 8.10 (d, J = 10.3 Hz, 1H), 7.77 (s, 1H), 7.63–7.61 (m, 2H), 7.52 (d, J = 8.8 Hz, 1H), 7.49–7.48 (m, 3H), 7.44 (d, J = 8.8 Hz, 1H), 1.49 (s, 9H).

Compound 13b

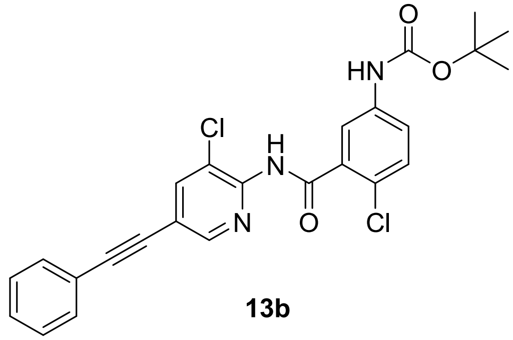

13b

***tert*-Butyl (4-chloro-3-((3-chloro-5-(phenylethynyl)pyridin-2-yl)carbamoyl)phenyl)carbamate (13b)**: Compound **13b** was synthesized in a similar manner to **14c**. Off-white powder (324 mg, 63% yield). m/z $[M+1]^+$: 482.2; LCMS retention time: 0.963 min (LCMS Method D); ^{1}H NMR (400 MHz, DMSO-d_6) δ 10.95 (s, 1H), 9.69 (s, 1H), 8.60 (d, J = 2.0 Hz, 1H), 8.30 (d, J = 2.0 Hz, 1H), 7.77 (d, J = 2.6 Hz, 1H), 7.64 – 7.58 (m, 2H), 7.53 (dd, J = 8.8, 2.7 Hz, 1H), 7.50 – 7.45 (m, 3H), 7.42 (d, J = 8.8 Hz, 1H), 1.48 (s, 9H). ^{13}C NMR (101 MHz, DMSO-d_6) δ 165.21, 152.66, 149.04, 147.22, 140.69, 138.58, 135.81, 131.59, 130.04, 129.52, 128.90, 126.43, 122.40, 121.38, 120.50, 118.67, 118.11, 93.28, 84.59, 79.68, 28.05. HRMS: Obs: 482.1036, Calcd: 482.1033 for $C_{25}H_{21}Cl_2N_3O_3$.

Compound 13c

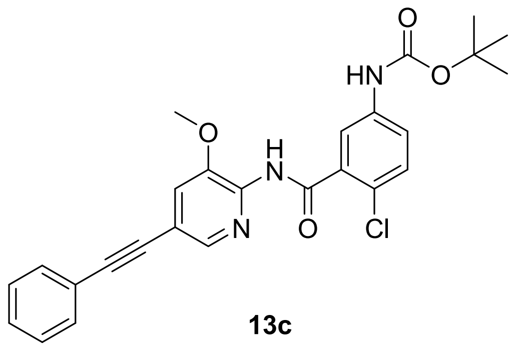

13c

***tert*-Butyl (4-chloro-3-((3-methoxy-5-(phenylethynyl)pyridin-2-yl)carbamoyl)phenyl)carbamate (13c)**: Compound **13c** was synthesized in a similar manner to **14c**. Off-white powder (30.8 mg, 65% yield). m/z $[M+1]^+$: 478.0; LCMS retention time: 0.889 min (LCMS Method D); ^{1}H NMR (400 MHz, DMSO-d_6) δ 10.36 (s, 1H), 9.65 (s, 1H), 8.14 (d, *J* = 1.8 Hz, 1H), 7.68 (dd, *J* = 6.0, 2.3 Hz, 2H), 7.65 – 7.55 (m, 2H), 7.50 (dd, *J* = 8.8, 2.7 Hz, 1H), 7.48 – 7.43 (m, 3H), 7.38 (d, *J* = 8.7 Hz, 1H), 3.88 (s, 3H), 1.48 (s, 9H). ^{13}C NMR (101 MHz, DMSO-d_6) δ 165.12, 152.66, 148.24, 141.43, 140.82, 138.43, 136.38, 131.45, 129.86, 129.17, 128.85, 122.41, 121.81, 121.56, 120.18, 118.15, 117.54, 91.76, 86.24, 79.63, 56.11, 28.05. HRMS: Obs: 478.1526, Calcd: 478.1528 for $C_{26}H_{24}ClN_3O_4$.

Compound 14a

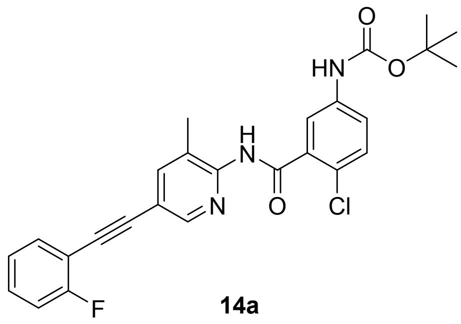

14a

***tert*-Butyl (4-chloro-3-((5-((2-fluorophenyl)ethynyl)-3-methylpyridin-2-yl)carbamoyl)phenyl)carbamate (14a)**: Compound **14a** was synthesized by the general procedures. m/z $[M+1]^+$: 480.4; LCMS retention time: 1.24 min (LCMS Method E); ^{1}H NMR (600 MHz, DMSO-d_6) δ 10.74 (s, 1H), 9.68 (s, 1H), 8.47 (s, 1H), 7.97 (s, 1H), 7.74 (s, 1H), 7.68 (t, J = 7.5 Hz, 1H), 7.54–7.51 (m, 2H), 7.42 (d, J = 8.8 Hz, 1H), 7.38 (t, J = 9.1 Hz, 1H), 7.31 (t, J = 7.6 Hz, 1H), 2.32 (s, 3H), 1.49 (s, 9H).

Compound 14b

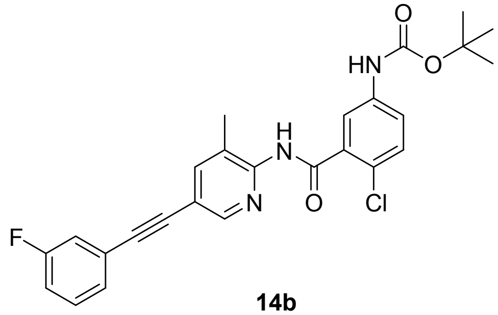

14b

***tert*-Butyl (4-chloro-3-((5-((3-fluorophenyl)ethynyl)-3-methylpyridin-2-yl)carbamoyl)phenyl)carbamate (14b)**: Compound **14b** was synthesized by the general procedures. m/z $[M+1]^+$: 480.4; LCMS retention time: 1.26 min (LCMS Method E); ^{1}H NMR (600 MHz, DMSO-d_6) δ 10.73 (s, 1H), 9.68 (s, 1H), 8.47 (s, 1H), 7.96 (s, 1H), 7.74 (s, 1H), 7.53–7.41 (m, 5H), 7.34 -7.31 (m, 1H), 2.32 (s, 3H), 1.49 (s, 9H).

IM-5 for Compound 14c

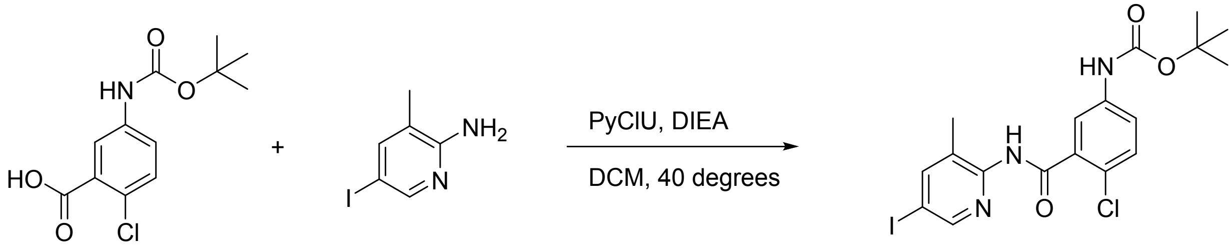

IM-5

***tert*-Butyl (4-chloro-3-((5-iodo-3-methylpyridin-2-yl)carbamoyl)phenyl)carbamate (IM-5)**: To a colorless solution of 5-(*tert*-butoxycarbonylamino)-2-chloro-benzoic acid (5.0 g, 18.4 mmol), 5-iodo-3-methyl-pyridin-2-amine (4.74 g, 20.2 mmol), DIEA (9.55 mL, 55.2 mmol) in DCM (50 mL) was added PyClU (9.18 g, 27.6 mmol). The reaction mixture was heated to 50 °C. After 1.5h, LCMS indicated product formation. The mixture was poured into $NaHCO_3$-*aq* and THF was added to dissolve solid materials. The mixture was extracted with ethyl acetate twice. The combined organic layer was washed with water, subsequently NH_4Cl-*aq*, water, brine and dried over $MgSO_4$. The filtrate was concentrated under reduced pressure to give a crude product, which was triturated with mixture of DCM (55.5mL) and hexane (55.5 mL) to yield **IM-5** as a white powder (7.11 g, 79% yield). m/z $[M+1]^+$: 488; ^{1}H NMR (400 MHz, DMSO-d_6) δ 10.61 (br, 1H), 9.67 (br, 1H),

8.50 (br, 1H), 8.14 (d, J = 1.5 Hz, 1H), 7.73 (d, J = 2.5 Hz, 1H), 7.52 (dd, J = 9.0, 2.5 Hz, 1H), 7.41 (d, J = 9.0 Hz, 1H), 2.26 (s, 3H), 1.49 (s, 9H).

Compound 14c = ONO-TR-772 / VU6018042

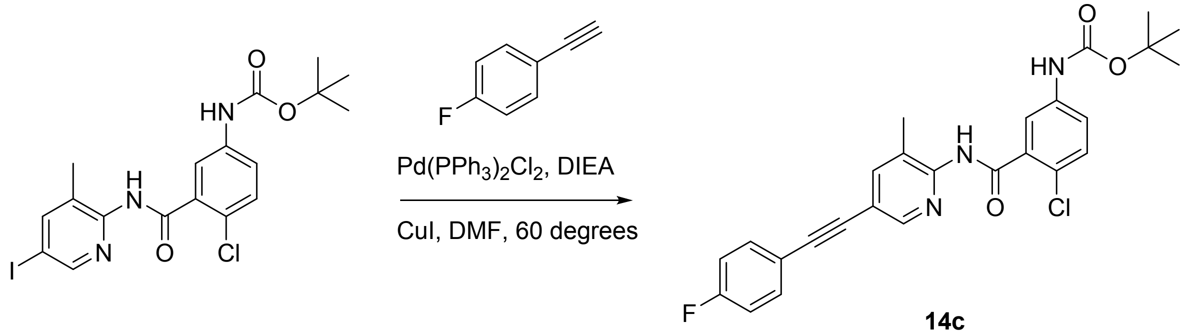

IM-5 **14c (ONO-TR-772 = VU6018042)**

***tert*-Butyl (4-chloro-3-((5-((4-fluorophenyl)ethynyl)-3-methylpyridin-2-yl)carbamoyl)phenyl)carbamate (14c)**: To a solution of **IM-5** (8.57 g, 17.6 mmol), 1-ethynyl-4-fluorobenzene (3.02 mL, 26.4 mmol), DIEA (30.4 mL, 175.8 mmol) in DMF (68 mL) was added CuI (167.4 mg, 0.88 mmol) and $Pd(PPh_3)_2Cl_2$ (616.9 mg, 0.88 mmol) under argon atmosphere. The reaction mixture was heated to 60 °C. After 3h, LCMS indicated product formation. The reaction mixture was diluted with ethyl acetate and poured into cold $NaHCO_3$-*aq* and THF was added to dissolve solid materials. The mixture was extracted with ethyl acetate / THF (c.a. 17 / 1) twice. The combined organic layers were diluted with hexane and washed with water, subsequently NH_4Cl-*aq*, water, brine and dried over $MgSO_4$. The organic layer was passed through Si-TMT and the filtrate was concentrated under reduced pressure to give a crude product, which was purified by column chromatography on silica gel and the concentrated residue was recrystallized with a mixture of THF, ethyl acetate and hexane to yield **14c** as a pale yellow powder (6.61 g, 78% yield). m/z $[M+1]^+$: 480.4; LCMS retention time: 1.25 min (LCMS Method E); ^{1}H NMR (600 MHz, DMSO-d_6) δ 10.70 (s, 1H), 9.66 (s, 1H), 8.44 (s, 1H), 7.92 (d, J = 1.8 Hz, 1H), 7.74 (s, 1H), 7.66–7.63 (m, 2H), 7.52 (dd, J = 9.0, 2.4 Hz, 1H), 7.40 (d, J = 8.4 Hz, 1H), 7.31–7.28 (m, 2H), 2.30 (s, 3H), 1.48 (s, 9H); ^{13}C NMR (150 MHz, DMSO-d_6) δ 165.23, 162.21 (d, J = 246.2 Hz), 152.65, 149.14, 148.05, 141.54, 138.57, 136.38, 133.83 (d, J = 9.2 Hz), 129.91, 128.83, 122.22, 120.23, 118.29 (d, J = 3.5 Hz), 117.95, 117.18, 116.11 (d, J = 21.9 Hz), 90.96, 85.72, 79.66, 28.03, 17.38;

HRMS: Obs: 480.1486, Calcd: 480.1485 for $C_{26}H_{23}ClFN_3O_3$ (HRMS Method (ii)); mp 200–203 °C.

Compound 14d

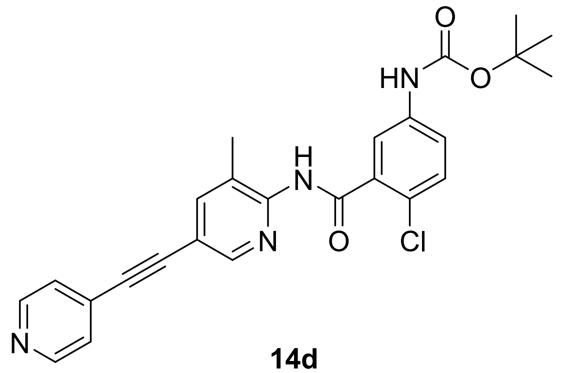

14d

***tert*-Butyl (4-chloro-3-((3-methyl-5-(pyridin-4-ylethynyl)pyridin-2-yl)carbamoyl)phenyl)carbamate (14d)**: Compound **14b** was synthesized by the general procedures. m/z $[M+1]^+$: 463.4; LCMS retention time: 0.96 min (LCMS Method E); ^1H NMR (600 MHz, DMSO-d_6) δ 10.77 (s, 1H), 9.68 (s, 1H), 8.66 (d, J = 4.6 Hz, 2H), 8.52 (s, 1H), 8.01 (s, 1H), 7.75 (s, 1H), 7.57 (d, J = 4.6 Hz, 2H), 7.53 (d, J = 8.8 Hz, 1H), 7.42 (d, J = 8.8 Hz, 1H), 2.32 (s, 3H), 1.49 (s, 9H).

Compound 15a

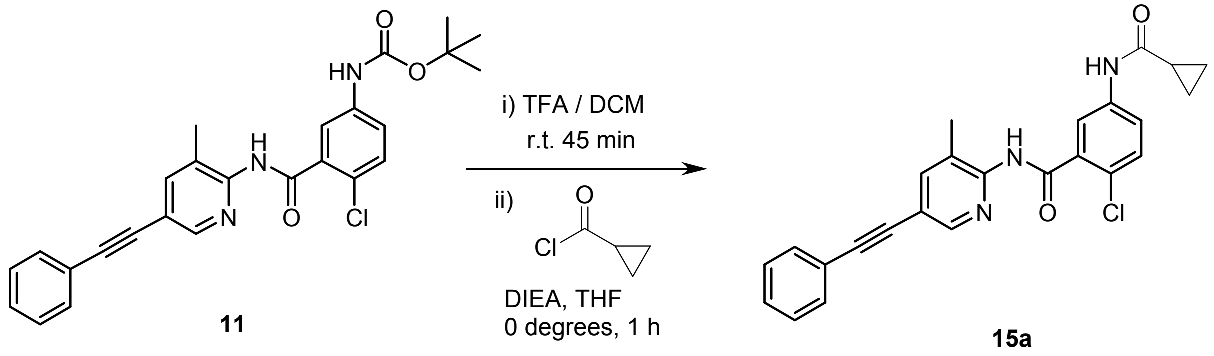

2-Chloro-5-(cyclopropanecarboxamido)-N-(3-methyl-5-(phenylethynyl)pyridin-2-yl)benzamide (15a): To a suspension of **11** (712 mg, 1.54 mmol) in DCM (7 mL) was added trifluoroacetic acid (7 mL) at ambient temperature. After the reaction mixture was allowed to stir for 45 minutes, the reaction mixture was concentrated under reduced pressure. $NaHCO_3$-*aq* was

added to the residue, and it was extracted with EtOAc twice. The combined organic layers were washed with $NaHCO_3$-*aq*, water, brine and dried over $MgSO_4$. The filtrate was concentrated under reduced pressure to give 5-amino-2-chloro-N-(3-methyl-5-(phenylethynyl)pyridin-2-yl)benzamide as a pale yellow powder, which was used for the next reaction without purification. To a solution of 5-amino-2-chloro-N-(3-methyl-5-(phenylethynyl)pyridin-2-yl)benzamide (400 mg, 1.11 mmol) in THF (6 mL) was added cyclopropanecarbonyl chloride (0.13 mL, 1.44 mmol) and *N,N*-diisopropylethylamine (0.38 mL, 2.21 mmol) at 0 °C. After 1h, LCMS indicated product formation. The reaction mixture was diluted with $NaHCO_3$-*aq* and extracted with EtOAc twice. The combined organic layers were washed with water, NH_4Cl-*aq*, brine and dried over $MgSO_4$. The filtrate was concentrated under reduced pressure to give a crude product (495 mg), which was triturated with EtOAc / Hexane (6 mL / 6 mL) to afford **15a** as an off-white powder (408 mg, 86% yield). m/z $[M+1]^+$: 430.3; LCMS retention time: 1.14 min (LCMS Method E); ^1H NMR (400 MHz, DMSO-d_6) δ 10.76 (s, 1H), 10.47 (s, 1H), 8.46 (s, 1H), 7.95 (d, J = 1.5 Hz, 1H), 7.89 (d, J = 2.5 Hz, 1H), 7.69 (dd, J = 8.8, 2.5 Hz, 1H), 7.61 – 7.59 (m, 2H), 7.48 – 7.46 (m, 4H), 2.32 (s, 3H), 1.78 (quintet, J = 6.1 Hz, 1H), 0.84 – 0.83 (d, J = 6.1 Hz, 4H).

Compound 15b

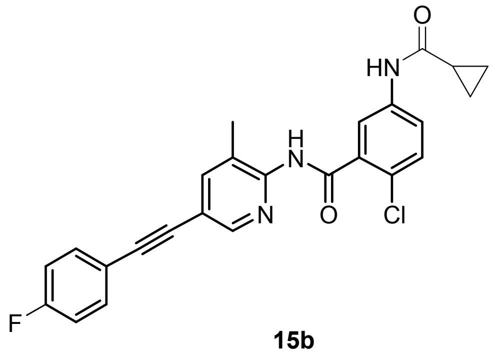

15b

2-Chloro-5-(cyclopropanecarboxamido)-N-(5-((4-fluorophenyl)ethynyl)-3-methylpyridin-2-yl)benzamide: Compound **15b** was synthesized in a similar manner to **15a**. m/z $[M+1]^+$: 448.3; LCMS Retention time: 1.15 min (LCMS Method E); ^1H NMR (600 MHz, DMSO-d_6) δ 10.74 (s, 1H), 10.48 (s, 1H), 8.46 (s, 1H), 7.94 (s, 1H), 7.89 (s, 1H), 7.70–7.65 (m, 3H), 7.46 (d, J = 8.6 Hz, 1H), 7.31 (t, J = 8.4 Hz, 1H), 2.31 (s, 3H), 1.78 (quint, J = 6.2 Hz, 1H), 0.84 (d, J = 6.2 Hz, 4H).

Characterization of 13b

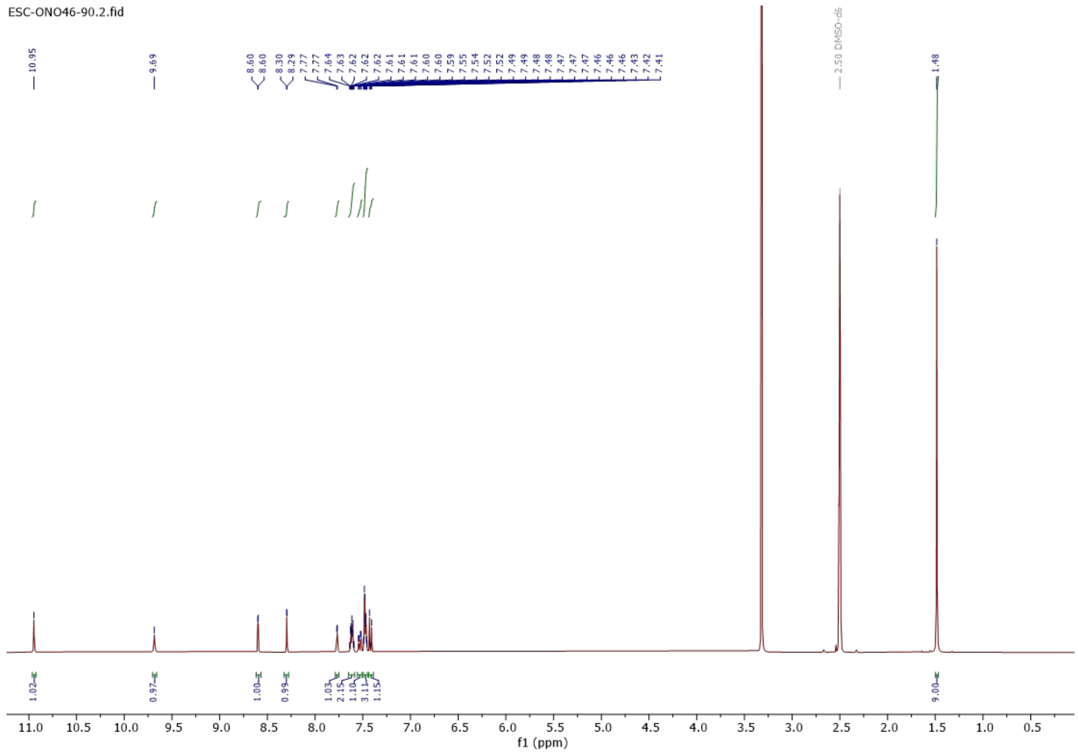

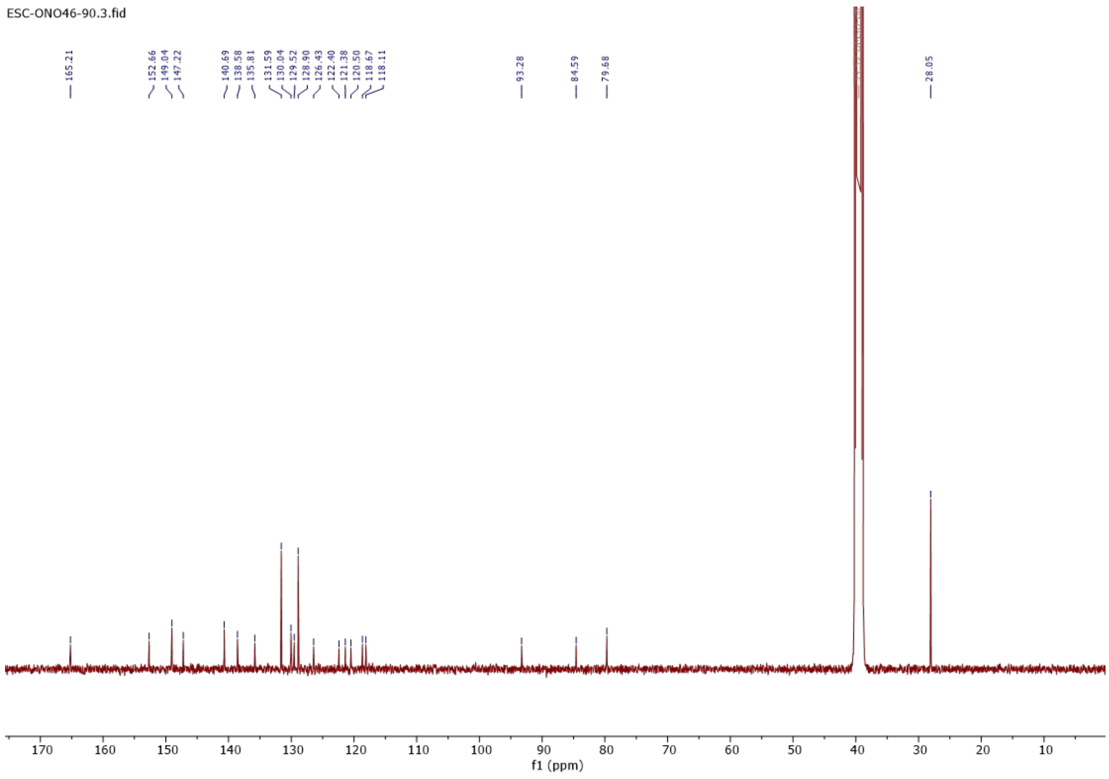

Characterization of 13c

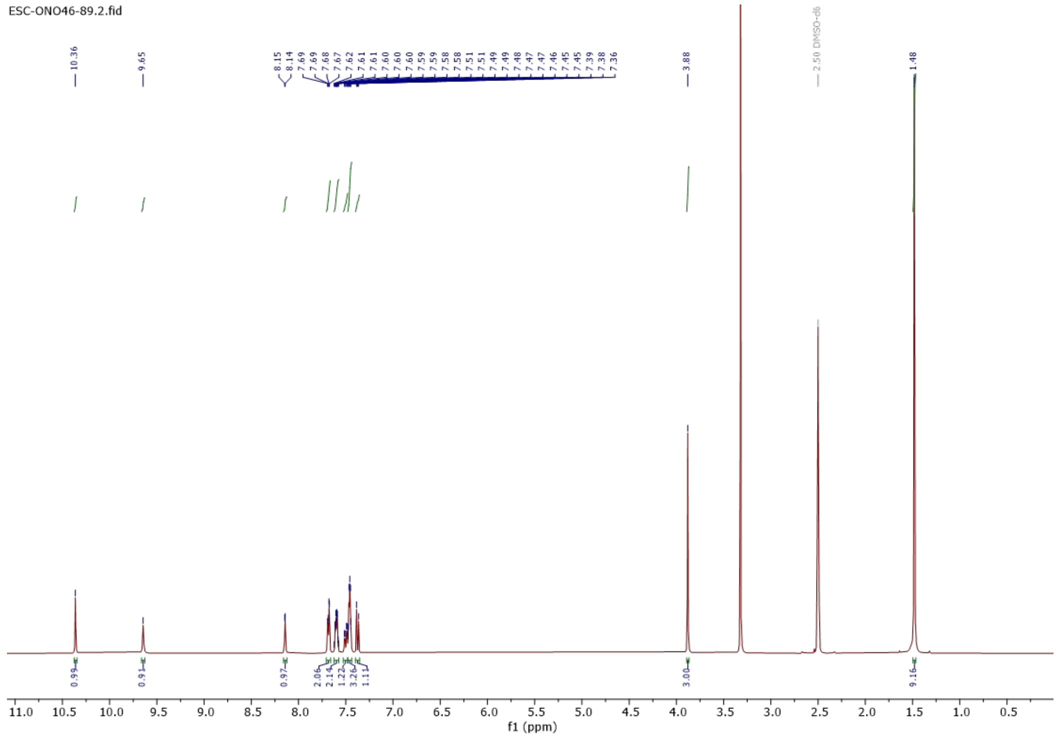

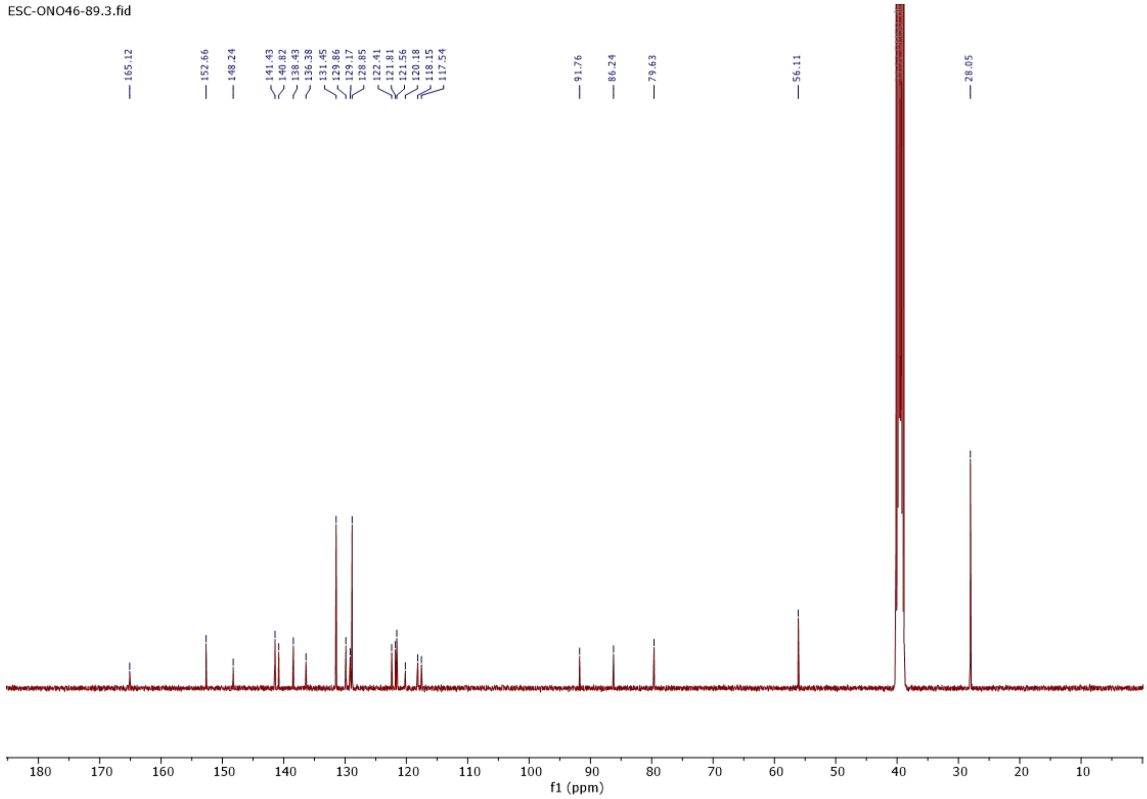

Characterization of 14c (ONO-TR-772 / VU6018042)

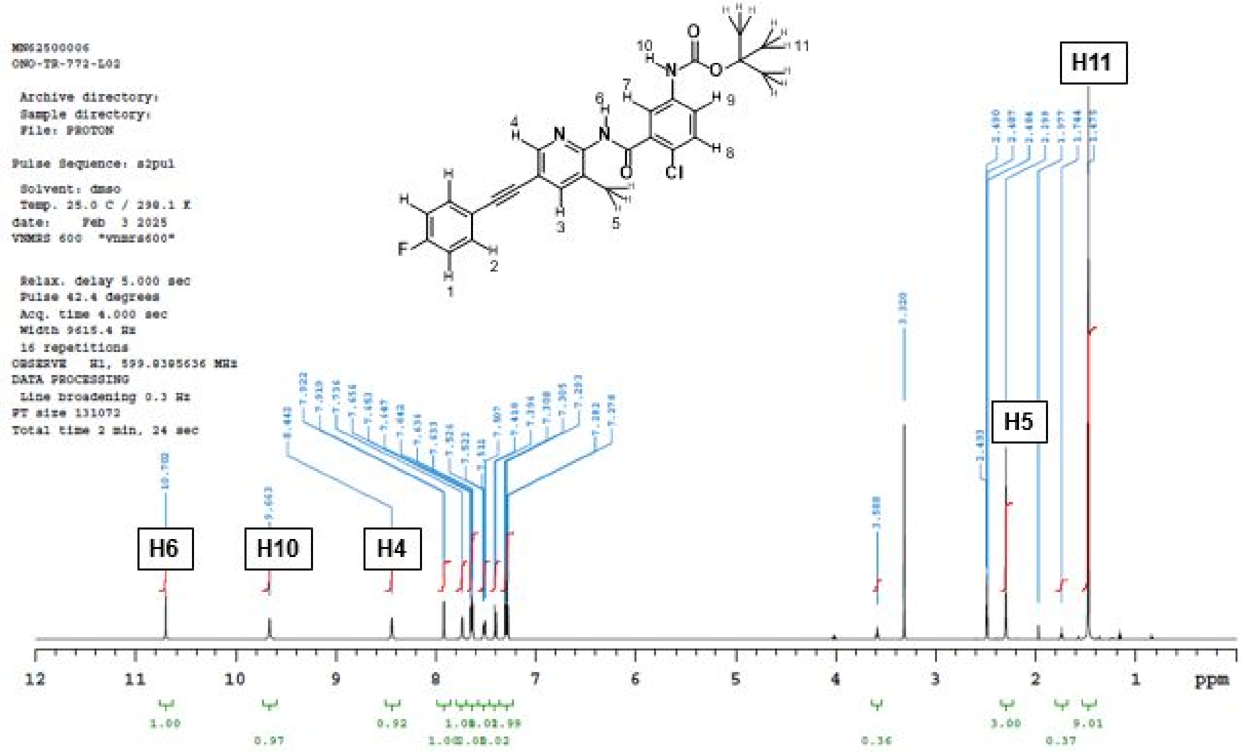

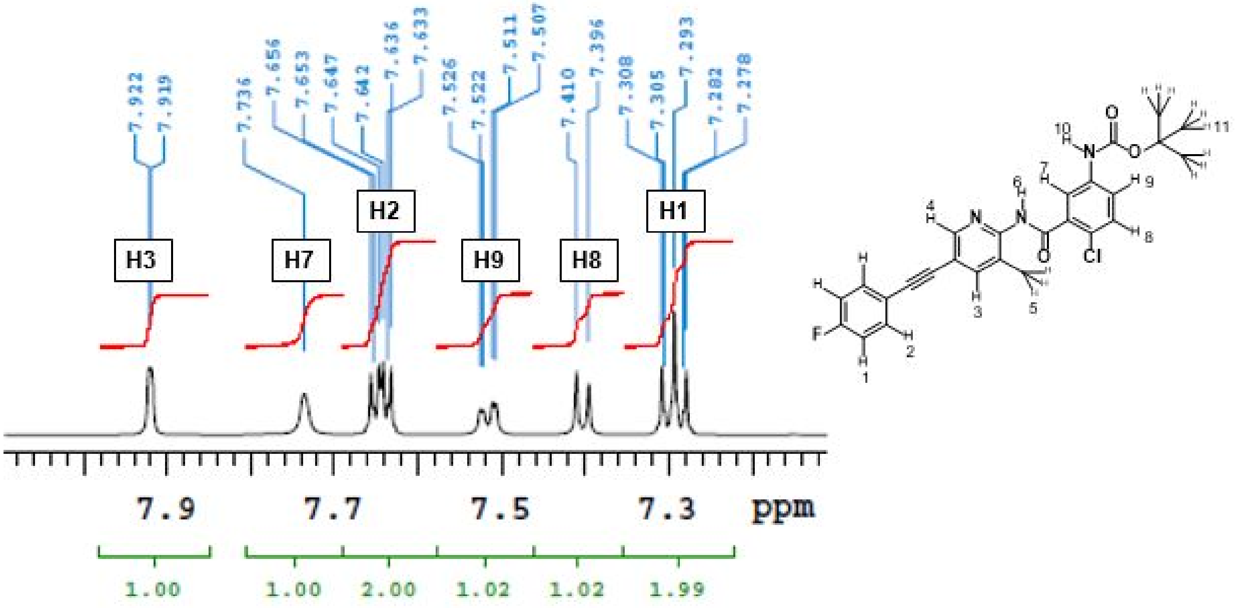

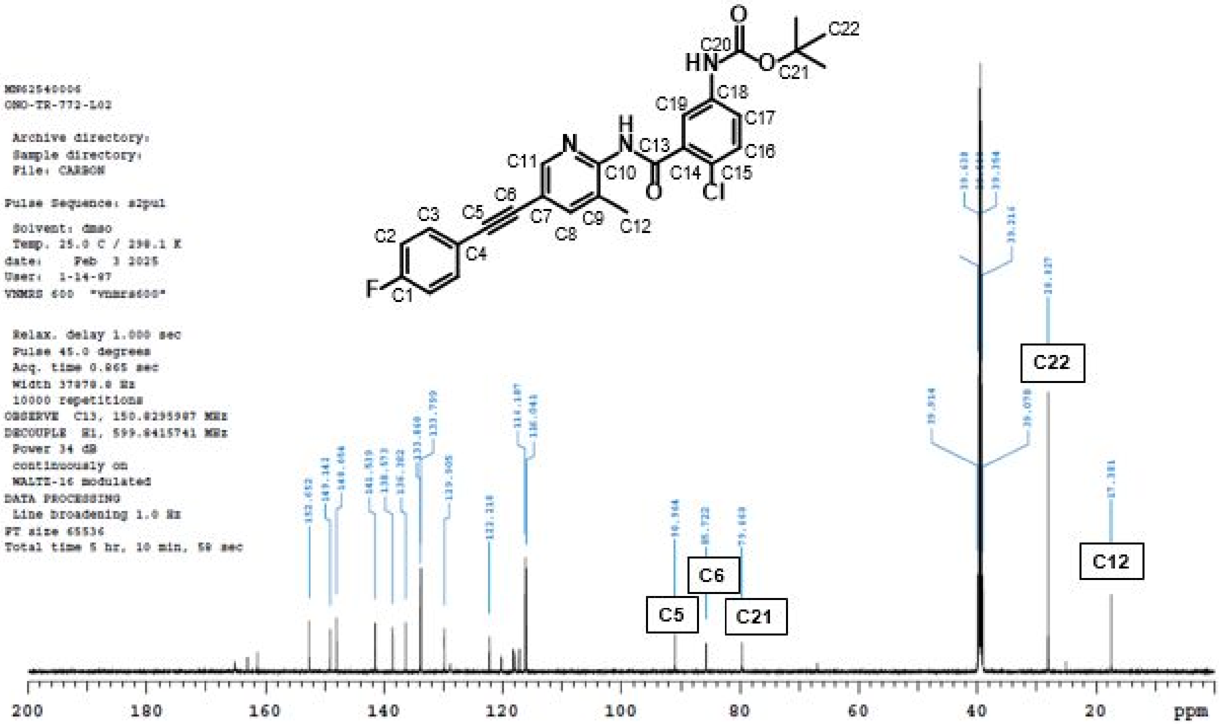
Archive directory:
Sample directory:
File: CARBON
Pulse Sequence: s2pul
Solvent: dmso
Temp. 25.0 C / 298.1 K
date: Feb 3 2025
User: 1-14-87
VNMRS 600 "vnmrs600"
Relax. delay 1.000 sec
Pulse 45.0 degrees
Acq. time 0.865 sec
Width 37878.8 Hz
10000 repetitions
OBSERVE C13, 150.8295987 MHz
DECOUPLE H1, 599.8415741 MHz
Power 34 dB
continuously on
WALTZ-16 modulated
DATA PROCESSING
Line broadening 1.0 Hz
FT size 65536
Total time 5 hr, 10 min, 59 sec
C22
C12
C5
C6
C21
200
180
160
140
120
100
80
60
40
20
ppm

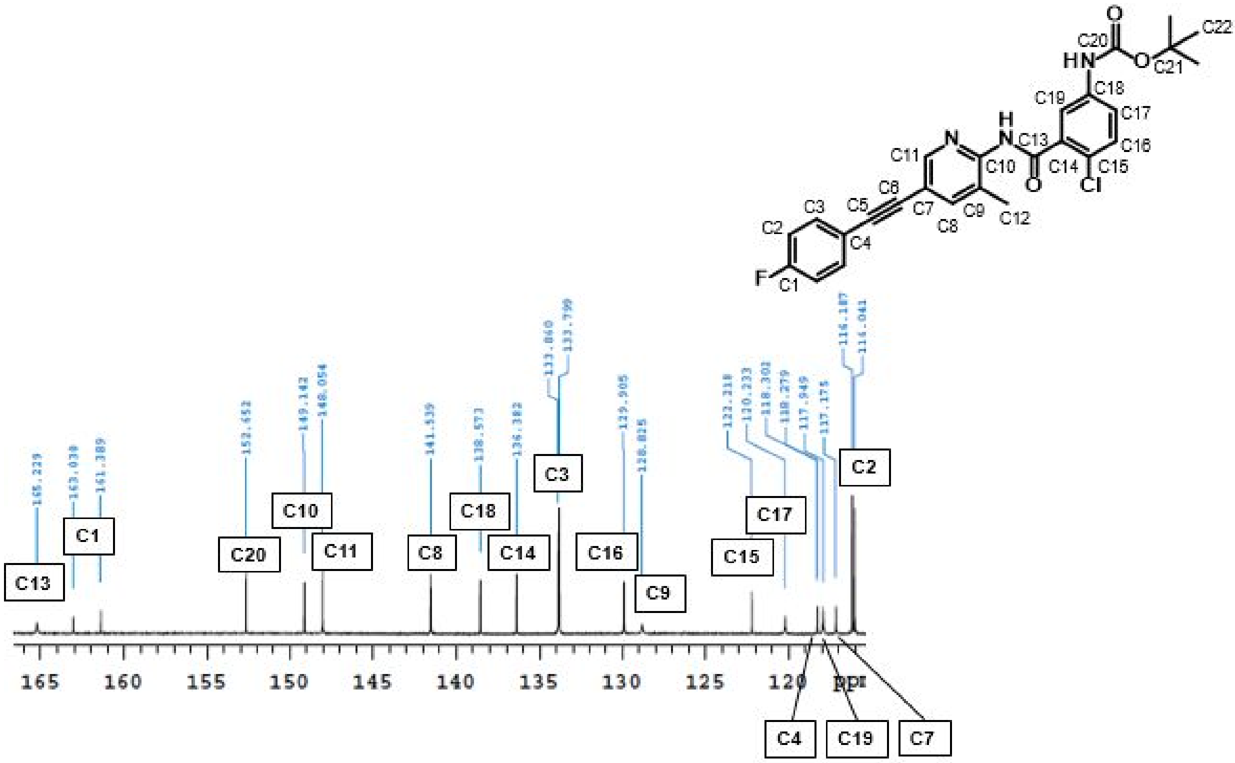
165.229
163.030
161.389
152.652
149.142
148.054
141.539
138.573
136.382
133.860
133.799
129.905
128.825
122.318
120.233
118.302
118.279
117.949
117.175
116.187
116.041
C13
C1
C20
C10
C11
C8
C18
C14
C3
C16
C9
C15
C17
C2
C4
C19
C7
165
160
155
150
145
140
135
130
125
120
ppm

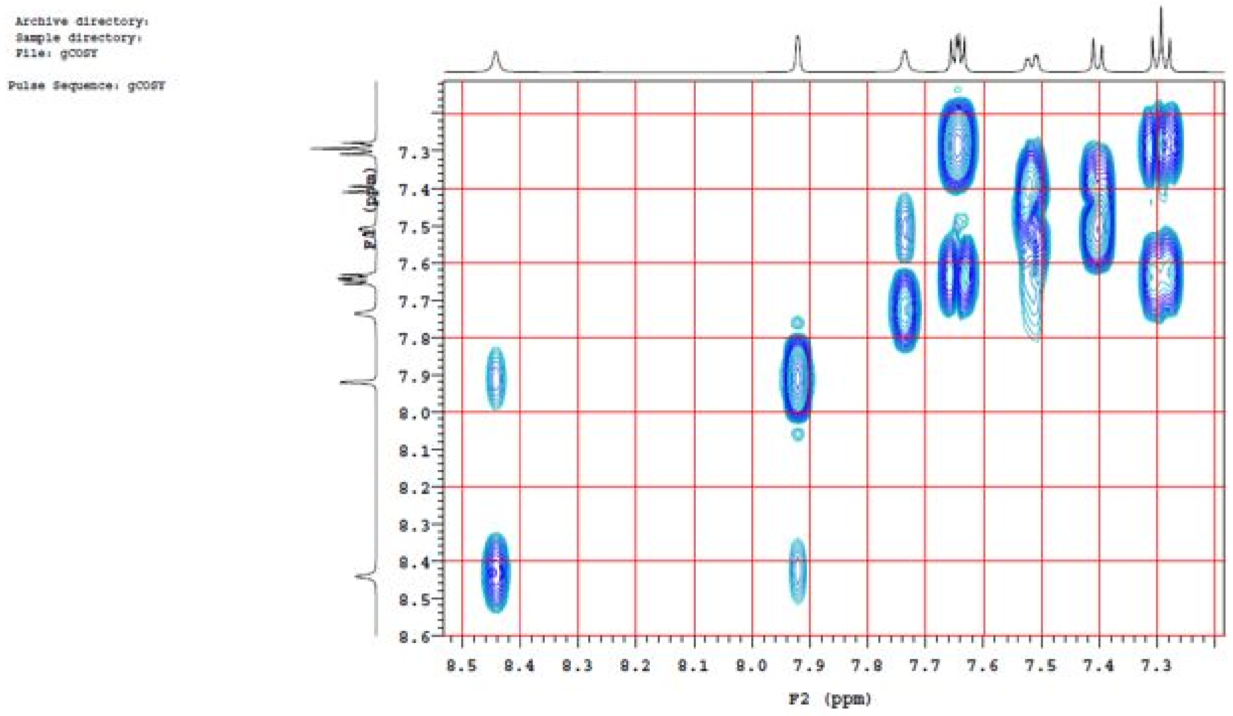
Archive directory:
Sample directory:
File: gCOSY
Pulse Sequence: gCOSY
F1 (ppm)
F2 (ppm)

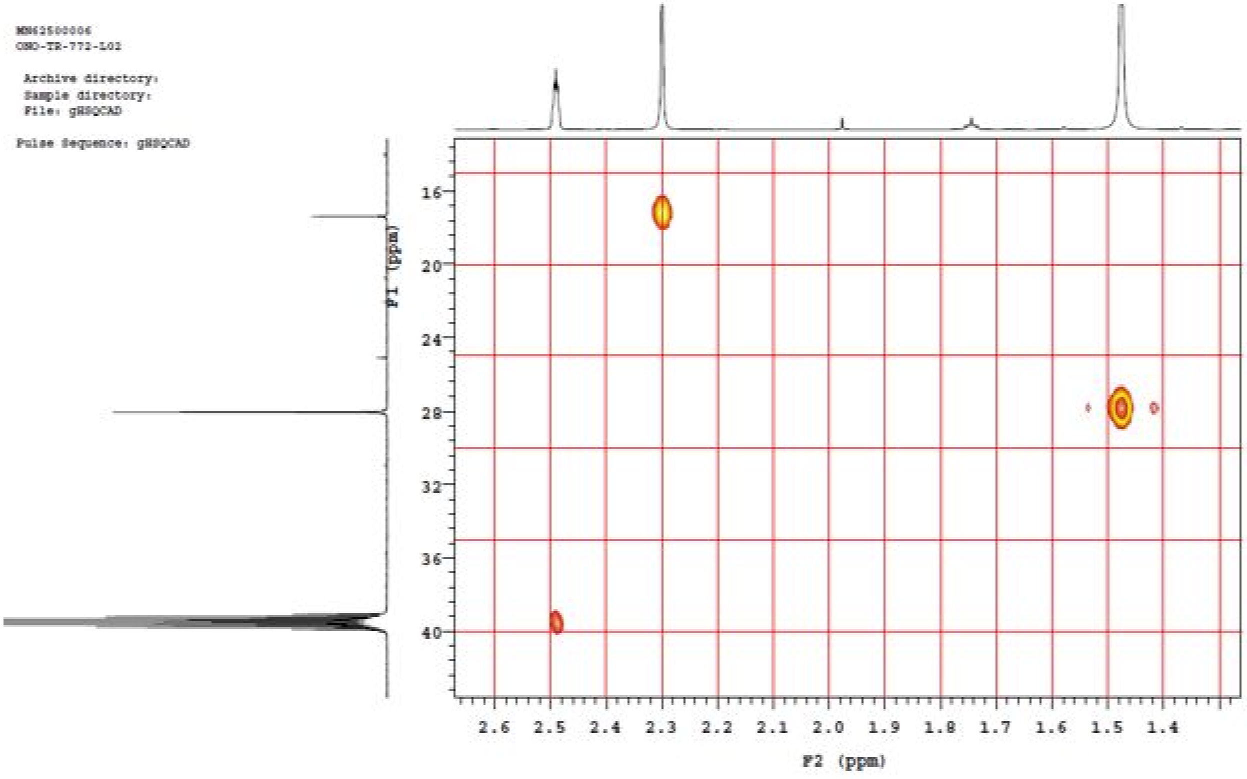
MN62500006
ONO-TR-772-L02
Archive directory:
Sample directory:
File: gHSQCAD
Pulse Sequence: gHSQCAD
F1 (ppm)
F2 (ppm)

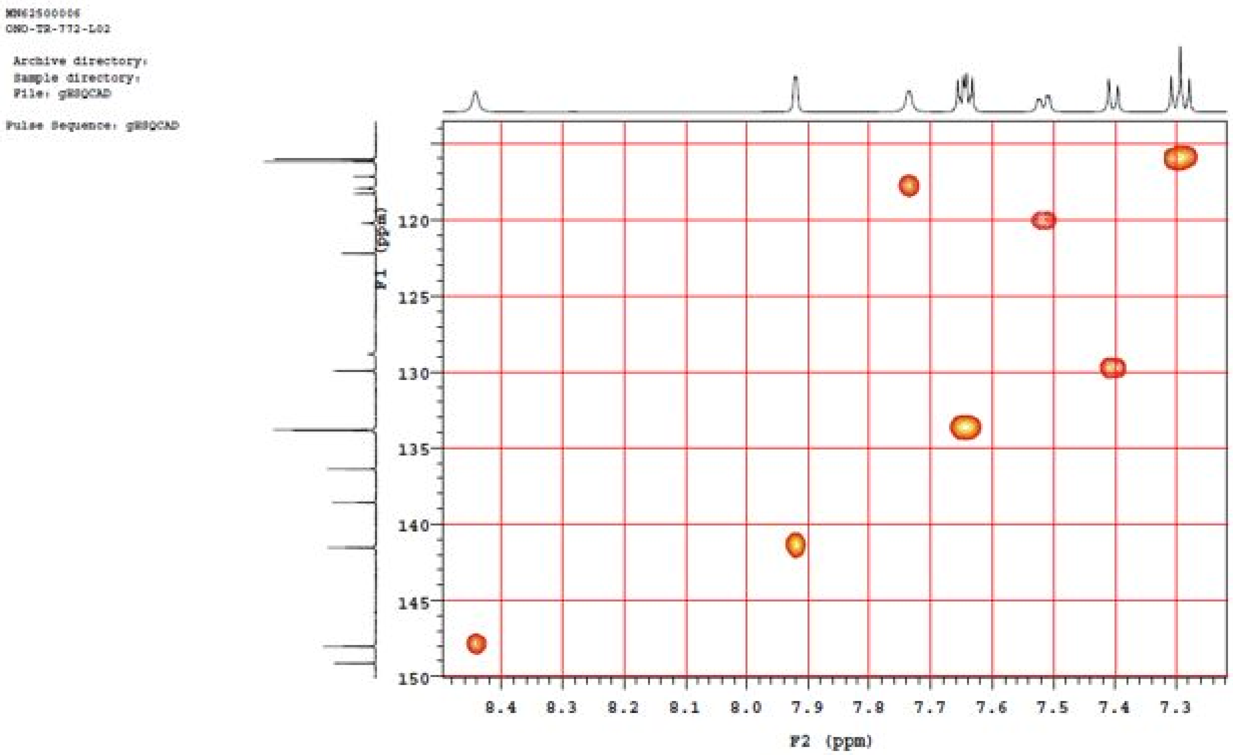

MN62500006
ONO-TR-772-L02
Archive directory:
Sample directory:
File: gHSQCAD
Pulse Sequence: gHSQCAD
F1 (ppm)
F2 (ppm)

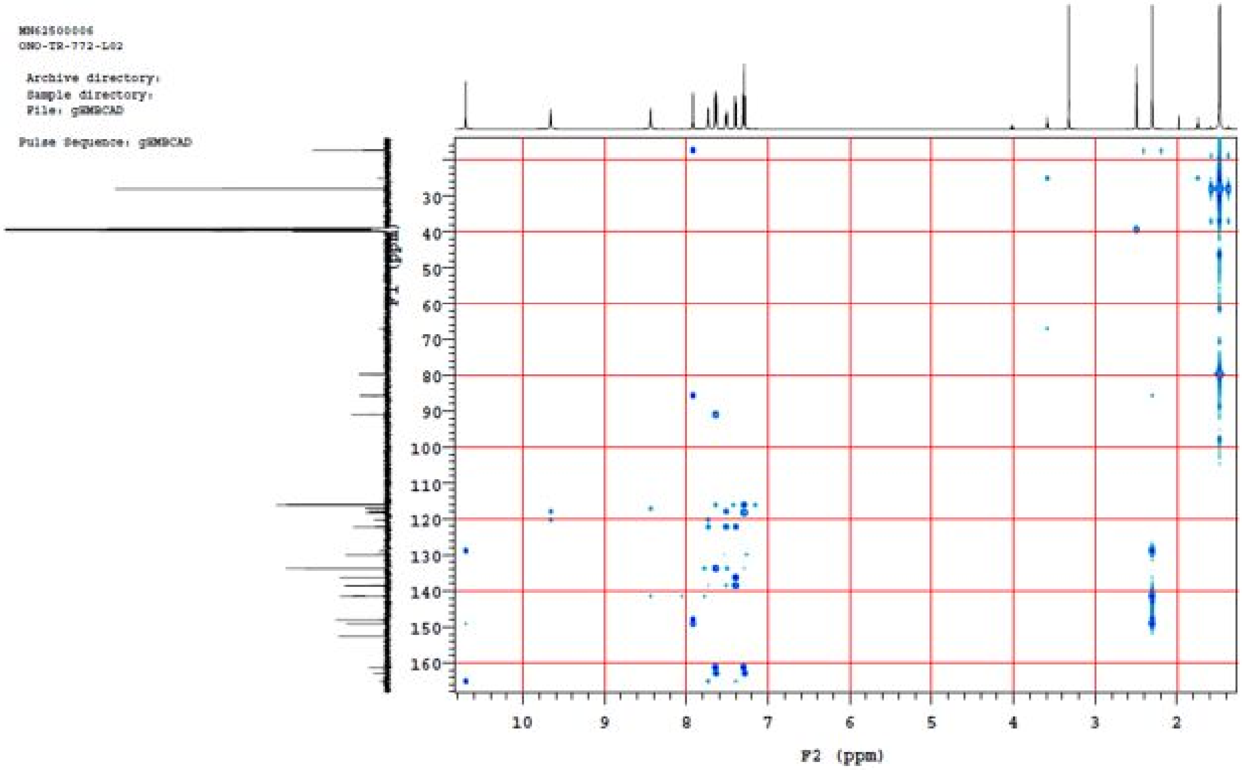

MN62500006
ONO-TR-772-L02
Archive directory:
Sample directory:
File: gHMBCAD
Pulse Sequence: gHMBCAD
F1 (ppm)
F2 (ppm)

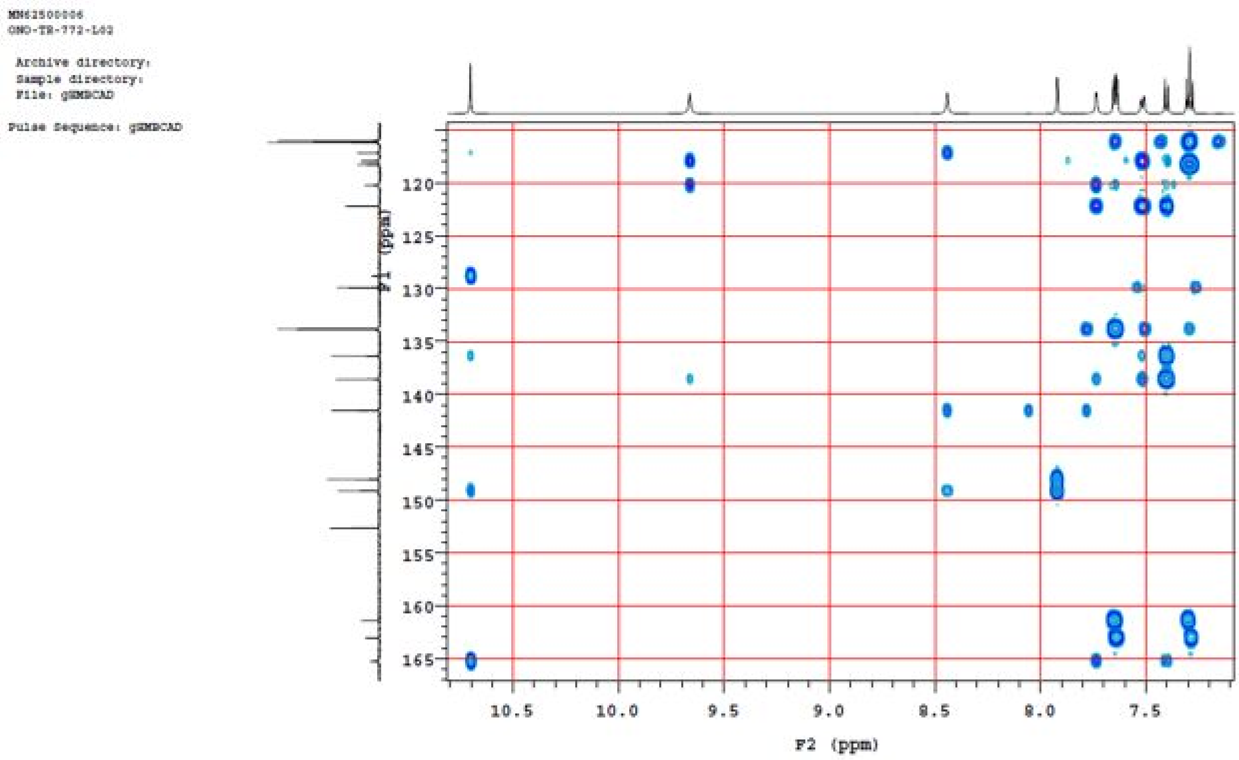
MN62500006
ONO-TR-772-L02
Archive directory:
Sample directory:
File: gHMBCAD
Pulse Sequence: gHMBCAD
F1 (ppm)
F2 (ppm)

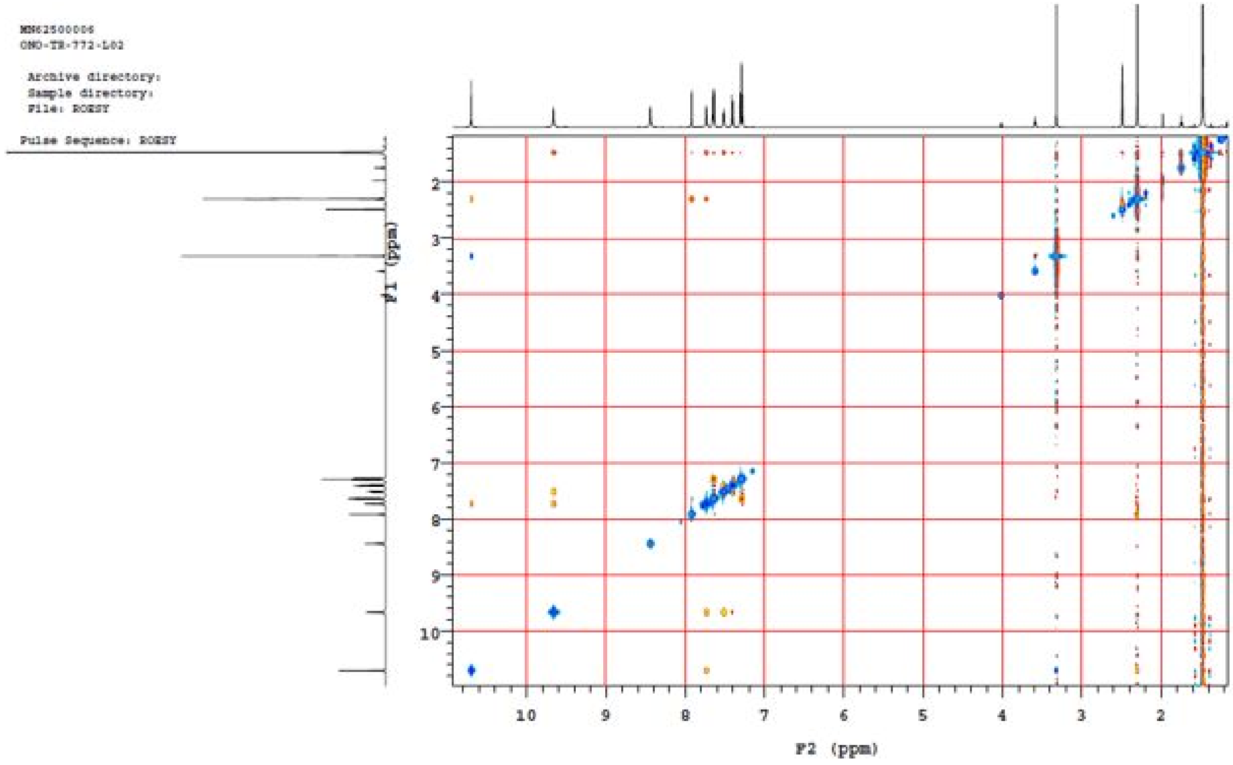
MN62500006
ONO-TR-772-L02
Archive directory:
Sample directory:
File: ROESY
Pulse Sequence: ROESY
F1 (ppm)
F2 (ppm)

ONO-TR-772 (mouse, p.o. 10mpk)

PK PBL of VU6018042-05 (ONO-TR-772) in Mouse After PO Administration

Project	Ono
Study	PK PBL
Animals	Mouse Male, CD-1 (*n* = 3-4 per time point; non-serially sampled)
Vehicle	10% Tween80 90% water (10 mL/kg; pH-adjusted with 1 N NaOH)
Dose	PO 10 mg/kg
Time points	0.25, 0.5, 1, 2, 4, 8, and 24 hr
Matrix	EDTA plasma; brain
Analyte	VU6018042-05 (ONO-TR-772)
MW	479.9
LLOQ	0.5 ng/mL

Dose (mg/kg)	Time (hr)	Concentration (ng/mL or g)				Mean Brain:Plasma K_p
		Plasma	Mean Plasma	Brain	Mean Brain	
10	0.25	19.6<br>47.1<br>64.4<br>77.0	52.0	2.44<br>3.22<br>7.64<br>12.9	6.56	0.13
	0.5	143<br>89.3<br>167<br>93.2	123	41.0<br>30.5<br>61.6<br>35.9	42.3	0.34
	1	206<br>193<br>221<br>198	205	68.6<br>79.2<br>82.8<br>66.4	74.3	0.36
	2	139<br>188<br>337	221	129<br>33.2<br>93.2	85.0	0.38
	4	350<br>237<br>184	257	186<br>80.5<br>97.1	121	0.47
	8	86.0<br>176<br>43.6	102	25.5<br>94.6<br>20.2	46.8	0.46
	24	17.4<br>15.0<br>16.6	16.3	8.48<br>5.29<br>7.75	7.17	0.44

PK Parameter	Unit	Plasma	Brain
Mean C_{max}	ng/mL or g	257	121
Mean T_{max}	hr	4	4
Mean $AUC_{0\text{-}inf}$	hr*ng/mL	n.d.	n.d.
Mean $AUC_{first\text{-}last}$	hr*ng/mL	2458	1089
Mean C_{max}	nM	536	252
Mean $AUC_{0\text{-}inf}$	hr*nM	n.d.	n.d.
Mean $AUC_{first\text{-}last}$	hr*nM	5122	2269

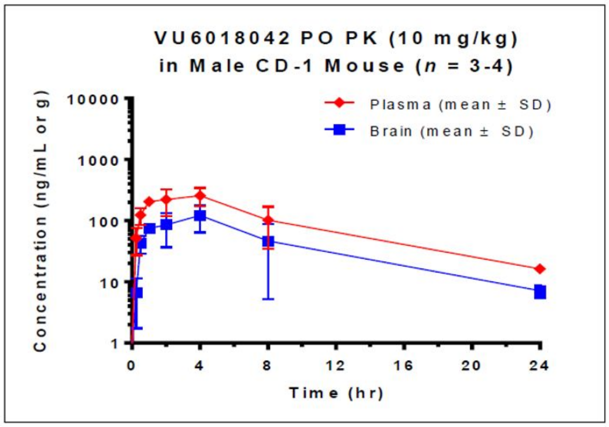

Figure S1. Mouse PO PBL PK at 10 mg/kg.

ONO-TR-772 (mouse, i.p. 10mpk)

PK PBL of VU6018042-05 (ONO-TR-772) in Mouse After IP Administration

Project	Ono
Study	PK PBL
Animals	Mouse Male, CD-1 (n = 2-4 per time point; non-serially sampled)
Vehicle	10% Tween80 90% water (10 mL/kg; pH-adjusted with 1 N NaOH)
Dose	IP 10 mg/kg
Time points	0.25, 0.5, 1, 2, 4, 8, and 24 hr
Matrix	EDTA plasma; brain
Analyte	VU6018042-05 (ONO-TR-772)
MW	479.9
LLOQ	0.5 ng/mL

Dose (mg/kg)	Time (hr)	Concentration (ng/mL or g) Plasma	Mean Plasma	Brain	Mean Brain	Mean Brain:Plasma K_p
10	0.25	460<br>18.9	239	74.3<br>36.1	55.2	0.23
	0.5	256<br>728	492	51.3<br>129	90.3	0.18
	1	1290<br>1090	1190	269<br>278	273	0.23
	2	1200<br>174	687	257<br>62.4	160	0.23
	4	849<br>1260<br>836<br>577	881	336<br>488<br>412<br>466	426	0.48
	8	532<br>701<br>289<br>542	516	181<br>292<br>208<br>412	273	0.53
	24	331<br>477<br>425<br>298	383	111<br>201<br>197<br>186	174	0.45

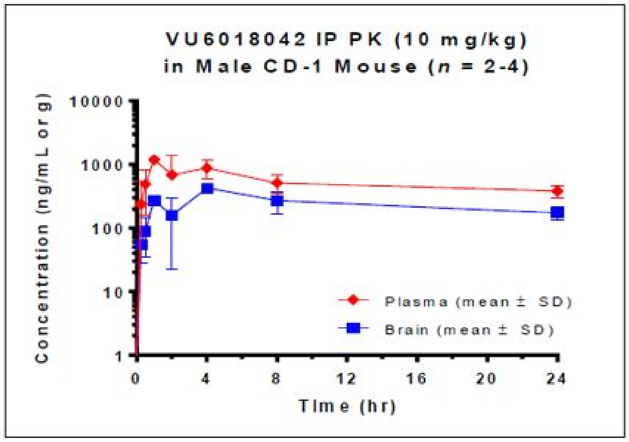

PK Parameter	Unit	Plasma	Brain
Mean C_{max}	ng/mL or g	1190	426
Mean T_{max}	hr	1	4
Mean AUC_{0-inf}	hr*ng/mL	n.d.	n.d.
Mean $AUC_{first-last}$	hr*ng/mL	13001	5884
Mean C_{max}	nM	2480	888
Mean AUC_{0-inf}	hr*nM	n.d.	n.d.
Mean $AUC_{first-last}$	hr*nM	27091	12261

Cmax (u,b): 5.3 nM based on mouse BHB of 99.4%
(a low estimate due to the lipophilicity of ONO-TR-772)

Figure S2. Mouse IP PBL PK at 10 mg/kg.

Modified Irwin Neurological Test Battery in Mice

	Vehicle					ONO-TR-772 30 mg/kg i.p.				
Time [hr]	1	2	4	8	24	1	2	4	8	24
Ptosis	0	0	0	0	0	0	0	0	0	0
Exophtalmus	0	0	0	0	0	0.33	0.17	0	0	0
Miosis	0	0	0	0	0	0	0	0	0	0
Mydriasis	0	0	0	0	0	0	0	0	0	0
Corneal reflex loss	0	0	0	0	0	0	0	0	0	0
Pinna reflex loss	0	0	0	0	0	0.17	0	0	0.20	0
Piloerection	0	0.33	0	0	0	0	0.17	0	0	0
Respiratory rate	0	0	0	0	0	0	0	0	0	0
Writhing	0	0	0	0	0	0	0	0	0	0
Tail erection	0	0	0	0	0	0	0	0	0	0
Lacrimation	0	0	0	0	0	0	0	0	0	0
Salivation	0	0	0	0	0	0	0	0	0	0
Vasodilation	0.17	0.17	0	0	0	0.17	0	0	0	0
Skin colour	0	0	0	0	0	0	0	0	0	0
Irritability	0	0	0	0	0	0	0	0.17	0	0
Somatomotor systems										
Motor activity	0	0	0	0	0	0	0.33	0	0	0
Convulsions	0	0	0	0	0	0	0	0	0	0
Arch/roll	0	0	0	0	0	0	0	0	0	0
Tremors	0	0	0	0	0	0	0	0	0	0
Leg weakness	0.17	0.33	0.17	0.17	0.17	0.33	0.17	0	0.20	0
Rigid stance	0	0	0	0	0	0	0	0	0	0
Spraddle	0	0	0	0	0	0	0	0	0	0
Placing loss	0	0	0	0	0	0	0	0	0	0
Grasping loss	0	0	0	0	0	0	0	0	0	0
Righting loss	0	0	0	0	0	0	0	0	0	0
Catalepsy	0	0	0	0	0	0	0	0	0	0
Tail pinch	0.33	0	0	0	0	0.33	0.50	0	0.20	0
Escape loss	0	0	0	0	0	0	0	0	0	0

The mean scores of 5-6 animals per treatment group
Modified Irwin scale: 0 = no effect, 1 = slight effect, 2 = pronounced effect.

Figure S3. Modified Irwin Neurological battery in mice at 30 mg/kg IP.
